# Microtubule lattice conformation and integrity regulate α-tubulin acetylation

**DOI:** 10.1038/s42003-026-10157-4

**Published:** 2026-06-08

**Authors:** Cornelia Egoldt, Marie-Claire Velluz, Joshua Tran, Charlotte Aumeier

**Affiliations:** Department of Biochemistry, University of Geneva, Geneva, Switzerland

**Keywords:** Microtubules, Cytoskeletal proteins

## Abstract

Microtubule acetylation of lysine 40 of α-tubulin is a hallmark of stable microtubules. This luminal modification is catalyzed by α-tubulin acetyltransferase 1 (αTAT1) and reversed by histone deacetylase 6 (HDAC6). However, acetylation regulation within the microtubule lumen and the influence of lattice architecture on enzymatic activity remain poorly understood. Here, we reconstitute microtubule acetylation in vitro using purified αTAT1 and HDAC6 on microtubules assembled with defined lattice conformations. We show that αTAT1 outweighs HDAC6 enzymatic activity, but its acetylation efficiency decreases upon microtubule damage. Importantly, αTAT1 efficiently acetylates microtubules with expanded lattices, while compacted lattices impede its activity. Our findings reveal that both microtubule integrity and lattice conformation are critical regulators for αTAT1 enzymatic activity, suggesting that dynamic transitions between compacted/expanded and intact/damaged lattices modulate the acetylation pattern of microtubules in cells.

## Abstract (web summary)

In vitro reconstitution shows microtubule acetylation is controlled by lattice structure. Expanded lattices promote αTAT1 activity-while compacted and damaged lattices impede it and favor HDAC6 deacetylation, linking structure to luminal modification.

## Introduction

Microtubules are dynamic cytoskeletal filaments that play key roles for cell shape, motility, and division^1^. Heterodimers of α- and β-tubulin binding guanosine triphosphate (GTP) assemble into polarized microtubule biopolymers, a hollow tube composed mainly of 13 protofilaments (PFs) in eukaryotic cells^2,3^. Within the microtubule lattice, tubulin can adopt different states: a guanosine diphosphate (GDP)-bound, compacted state lattice and an expanded, GTP-bound state at the microtubule tip. The compacted GDP-microtubule lattice is under more strain, with an average dimer rise of 81.5 Å within a protofilament, compared to an expanded dimer conformation of 83.5–84 Å in the GTP-cap^4–8^.

The level of lattice expansion can be read and/or modulated by different microtubule-associated proteins (MAPs): EB3 or DCX bind to the microtubule surface at the interface of 4 tubulin dimers^9,10^, TPX2 binds across longitudinal and lateral protofilaments^11^ and MAP7 and tau bind longitudinally within a protofilament^12,13^. Thereby, they reshape the biochemical landscape of microtubules, locally fine-tuning microtubule function and stability.

In vitro, by replacing the nucleotide GTP with its slowly hydrolysable analogue guanylyl-(α,β)-methylene-diphosphate (GMPCPP), stable microtubules nucleate with 14 protofilaments in an expanded lattice state (84 Å)^4,6,8,14^. In addition, specific lattice conformational states can be generated in vitro by using different microtubule-stabilizing agents (MSAs). Two distinct ligand-binding sites on β-tubulin exist for MSAs, the taxane site and the laulimalide/peloruside site, both preventing microtubule depolymerization^15^.

Paclitaxel (taxol) has been shown to expand the microtubule lattice inducing a GTP-like state (82.3 Å), with a preference for 13-PF microtubules^4,5,16,17^. The taxane site is in the microtubule lumen, and taxol binding has been reported to stabilize β-tubulin in a conformation that favors lateral contacts^18–20^. Furthermore, simulations showed that taxol exerts a “pseudo” kinetic stabilization through decreasing the strain energy of the GDP-tubulin lattice^21^. The laulimalide/peloruside site is located on the outside of the microtubule near the lateral interface of protofilaments^15^. Structural analysis showed that peloruside A acts on heterologous lateral tubulin contacts at the microtubule lattice seam, a discontinuity in the microtubule lattice^5,22^. Through binding to β-tubulin it supports a homologous, non-seam interaction. Opposite to taxol, peloruside A does not induce microtubule lattice expansion but rather compacts the lattice (81.0 Å) and even overrides the effects of taxol binding^5^.

In cells, microtubules are dynamic and exposed to different stresses like mechanical forces, the activity of motor proteins or severing enzymes, all potentially removing tubulin dimers from the shaft^23–26^. Dimer dissociation can occur spontaneously along the microtubule shaft, as well as at a switch in protofilament number or at a transition in the helix pitch^27,28^. Changes in seam number and seam location can also leave holes in the lattice during microtubule assembly^28,29^. This damage can be repaired to rescue microtubule growth by the incorporation of free GTP-tubulin into the lattice^30–32^.

Previous results from our group and others showed that the processive motor protein kinesin-1 induces shaft damage^24,33,34^. Kinesin-1 motors generate force while running along a protofilament, which results in dimer dissociation from the shaft^35–38^. Furthermore, kinesin-1 has been shown to preferentially bind to GTP-tubulin within the lattice, to buckle microtubules and to expand the microtubule lattice^39–42^. A 5-amino acid deletion in the neck linker region results in a kinesin-1 mutant that damages microtubules more severely while running along them^34^.

In the cellular network, microtubules are highly modified by MAPs which add and remove post-translational modifications (PTMs) to tubulin subunits in a tightly regulated manner. Modifications like de-/acetylation, de-/tyrosination or glutamylation determine the “tubulin code” of specific microtubule subsets^43,44^.

Tubulin acetylation is a modification that occurs on the lysine 40 (K40) residue of α-tubulin, which resides in the microtubule lumen upon microtubule polymerization^45,46^. The level of K40 acetylation in the mammalian brain is about 30%^47^. The enzyme α-tubulin acetyl transferase 1 (αTAT1) acetylates tubulin nearly exclusively once it is polymerized into microtubules and must therefore access the lumen for K40 modification^48,49^. Hence, αTAT1 enters through open ends and through damage sites along the microtubule shaft to diffuse and stochastically acetylate α-tubulin^48,50,51^. In cells, this acetylation behavior leads to distinct acetylation segments along a single microtubule^52^. HDAC6 is three times larger in size (140 kDa) compared to αTAT1 (45 kDa)^49,53^ and deacetylates tubulin with a 1500-fold higher rate than polymerized microtubules^54,55^.

In vitro experiments demonstrated that αTAT1 acetylates and HDAC6 deacetylates microtubules stochastically along their entire length^48,54^. In cells, microtubule acetylation forms a gradient, with high acetylation around the nucleus decreasing towards the cell periphery^52^. This gradient results from a tightly controlled interplay between αTAT1 and HDAC6. We recently showed that running kinesin-1-induced microtubule damage changes the cellular acetylation gradient, reduces cell acetylation levels and results in deacetylation specifically around damage sites^52^. But whether both enzymes counteract each other within the microtubule lumen to modify the K40 residue on α-tubulin and how microtubule lattice conformation impacts enzymatic activity remains unknown.

In this study, we test microtubule acetylation in a reconstituted model system. We use purified αTAT1 and HDAC6 proteins on in vitro assembled microtubules. We show that αTAT1 outcompetes HDAC6 enzymatic activity but that αTAT1 acetylation efficiency decreases when microtubules are damaged. We demonstrate that not only loss of microtubule integrity but also the state of the underlying microtubule lattice impacts acetylation efficiency, as αTAT1 prefers to acetylate microtubules in an expanded state.

## Results

### αTAT1 outweighs HDAC6 activity on intact microtubules in vitro

**Fig. 1:**
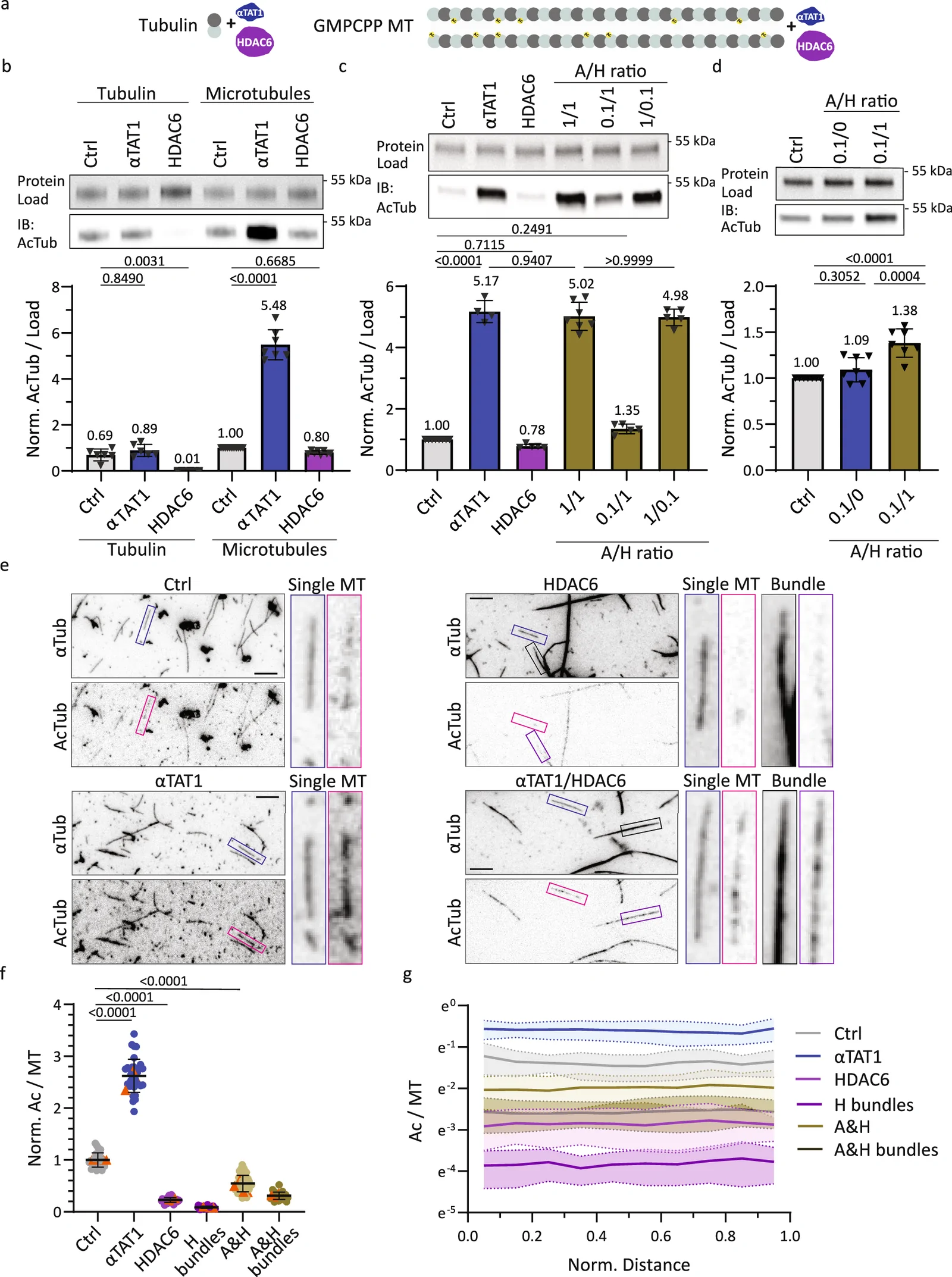
αTAT1 activity is high and HDAC6 activity low on intact microtubules in vitro. **a** Cartoon showing the in vitro reconstitution setup for unpolymerized tubulin or GMPCPP-microtubules with αTAT1 and HDAC6. Representative Western blot analysis for (de)acetylation reactions with **b** αTAT1 (1 µM) and HDAC6 (1 µM) in presence of tubulin (1 µM) or GMPCPP-microtubules (1 µM), **c** for GMPCPP-microtubules (1 µM) with αTAT1 (1 µM), HDAC6 (1 µM), or different αTAT1/HDAC6 ratios (A/H 0.1 or 1 µM) and **d** with 0.1 µM αTAT1 in the absence or presence of HDAC6 (1 µM). Quantifications of acetylated tubulin (AcTub) levels are normalized to protein load, from 5–7 (**b**) or 4–7 (**c**, **d**) independent experiments. Statistics: one-way ANOVA. Mean with SD. **e** Representative immunofluorescence images of microtubules (MT) that were antibody stained for acetylated tubulin; Scale bars: 5 µm. The boxed areas show zoom-ins for single and bundled microtubules; identical settings of brightness and contrast were used to visualize differences in acetylation levels. **f**, **g** Acetylation levels over microtubule fluorescence for Ctrl (*n* = 25–30), αTAT1 (*n* = 29–33), HDAC6 (*n* = 27), HDAC6 (H) bundles (*n* = 30), αTAT1 and HDAC6 (A&H 1:1; *n* = 40–45) or αTAT1 and HDAC6 (A&H) bundles (*n* = 30), from 3–4 independent experiments. In **f**, the normalized (Norm.) average acetylation signal (orange triangle: experimental mean) per microtubule is shown and in **g**, acetylation signals are plotted over the normalized (Norm.) distance of each microtubule. For individual data, see Supplementary Fig. 3d. Statistics: one-way ANOVA. Mean with SD.

Confirming previous findings^48,54^, our in vitro reconstitution assay shows that HDAC6 preferentially deacetylates non-polymerized brain tubulin and αTAT1 preferentially acetylates preformed microtubules (GMPCPP-stabilized). Notably, microtubule length does not affect αTAT1 acetylation efficiency^56^. Within 60 min, HDAC6 deacetylates brain tubulin nearly completely, while reducing polymerized microtubule acetylation levels by only 20% compared to control conditions (Fig. 1a–c). αTAT1, on the other hand, barely acts on free tubulin but increases microtubule acetylation levels by 5.5-fold within 60 min (Fig. 1b). Under these conditions, acetylation levels approach saturation, and increasing αTAT1 concentration does not further enhance tubulin acetylation (Supplementary Fig. 1a).

When both enzymes are present in the reaction at a 1:1:1 ratio with polymerized tubulin, αTAT1 shades the low deacetylation activity of HDAC6 and acetylation increases fivefold despite the presence of HDAC6 (Fig. 1c). Given that a cell expresses HDAC6 with an approximately 6× fold higher protein copy number than αTAT1^57^, we next reduced the amount of αTAT1 in the reaction. Lowering the amount of αTAT1 by tenfold while keeping the HDAC6 and tubulin concentration constant, still shifted the system towards a 1.35-fold acetylation increase compared to control conditions. By contrast, αTAT1 alone at this low concentration was insufficient to acetylate microtubules within 60 min, suggesting that HDAC6 enhances αTAT1-mediated acetylation at low αTAT1 concentrations (Fig. 1d). This enhancement may arise from indirect effects, like on the microtubule substrate (e.g., bundling or lattice effects; see below) or from direct interaction with αTAT1. Lowering HDAC6 by tenfold while keeping tubulin and αTAT1 stable at a higher concentration shows no impact on acetylation efficiency (Fig. 1c). Hence, HDAC6 shows low deacetylation activity towards microtubules but enhances αTAT1 activity at low αTAT1 concentrations. At higher αTAT1 concentrations (>0.5), αTAT1 activity becomes independent of the presence of HDAC6 (Supplementary Fig. 1a).

To visualize the acetylation level along GMPCPP-stabilized microtubules, we used antibody staining. Immunofluorescence reveals a 2.6-fold increase in acetylation in the presence of αTAT1 (Fig. 1e, f), which is lower but consistent with our Western blot data. In contrast, HDAC6 treatment leads to a deacetylation of 80%, substantially more than observed by Western blot. When both enzymes, αTAT1 and HDAC6, are present at a 1:1:1 ratio with tubulin, the antibody showed a 40% decrease in acetylation, whereas Western blotting shows an increase (Fig. 1e, f). These discrepancies suggest that the antibody is less efficient in recognizing acetylation levels by immunofluorescence in vitro than by Western blot.

In addition, HDAC6 not only acts as a deacetylase but also promotes microtubule bundling (Supplementary Fig. 1b). Bundled microtubules in HDAC6 or αTAT1/HDAC6 conditions show even lower acetylation signals compared to single microtubules (Fig. 1e, f). Furthermore, the acetylation signal is inhomogeneous when microtubules were treated with HDAC6, resulting in patches along the microtubule shaft (Fig. 1e), consistent with previous findings by Soppina et al.^45^. Quantitatively, this patchiness was reflected in a higher standard deviation of fluorescence intensity compared to control or αTAT1-only conditions (Fig. 1g, and Supplementary Fig. 3d). The acetylation reduction and its patchiness is not due to general steric hindrance for antibodies due to HDAC6 binding to microtubules, as α-tubulin staining remained unaffected (Supplementary Fig. 1c). However, it is possible that HDAC6 interferes with antibody access to the luminal epitope. Given prior in vitro findings showing that taxol disrupts antibody recognition^45^ (Supplementary Fig. 1d) and considering that additional lattice inhomogeneities (e.g., lattice expansion) may further impair this antibody to bind, we decided to base our quantitative analysis primarily on Western blotting.

### Characterization of microtubule damage

**Fig. 2:**
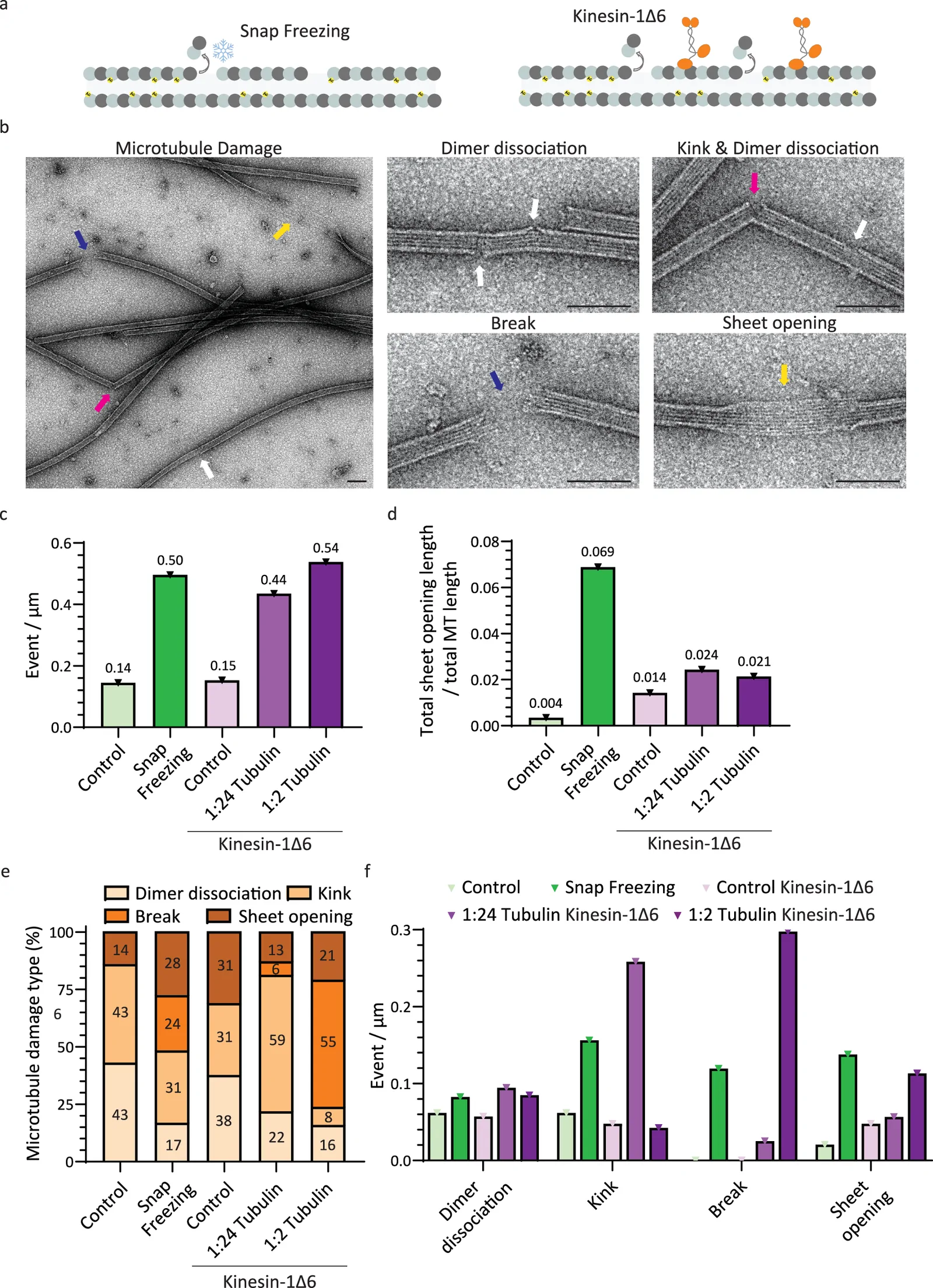
Diversity and abundance of microtubule damage. **a** Cartoons displaying microtubule damage generation in vitro per either snap freezing (left) or running kinesin-1Δ6-induced damage (right; more details see “Methods”). **b** Representative Electron Microscope (EM) image of different damage types of GMPCPP-stabilized microtubules incubated with kinesin-1Δ6 in a 1:24 tubulin ratio. Damage types are indicated with color-coded arrows: magenta - kink, white **-** dimer dissociation, yellow **-** sheet opening, blue - break with microtubule ends <100 nm apart. Zoom-ins are shown for the different damage types indicated with the same color-coding. Bundles were excluded from analysis. Scale bars: 100 nm. **c** Damage frequency plotted as event per micrometer (total microtubule length) for all conditions. Note that control conditions for kinesin treatment and snap freezing differed in incubation; details are provided in the “Methods”. **d** Fraction of total microtubule length in an open-sheet configuration (sum of sheet-opening lengths divided by total microtubule length), based on EM quantification for all conditions. **e** Distribution (%) of different damage types per condition. **f** Damage frequency as event per micrometer of different damage types for all damaging conditions.

In cells, microtubules can get damaged through different mechanisms, like mechanical strain, motor proteins, or microtubule severing enzymes^24,25^. To reconstitute microtubule damage in vitro, we sought to induce damage artificially using two different methods (Fig. 2a). First, “Snap freezing”, where we performed 5 cycles of snap freezing followed by quick thawing of GMPCPP-stabilized microtubules to induce damage to microtubules (see “Methods” for more details). Second, to induce more physiologically relevant damage, we purified the highly damaging kinesin-1 mutant, kinesin-1Δ6^34^, and incubated it with GMPCPP-stabilized microtubules, as wild-type kinesin-1 damages stabilized microtubules insufficiently^34^. To characterize microtubule damage induced by the two methods, we used negative stain electron microscopy (EM). We categorized observed damages into four classes of increasing severity: dimer dissociation, kink, break and sheet opening (Fig. 2b, and Supplementary Fig. 2a). Dimer dissociation and kinks typically involve the loss of a few dimers from the lattice (Supplementary Fig. 2b), though kinks likely reflect a more extensive local disruption. Potential kinks could result in breakage, where the new resulting ends should be in proximity. Sheet openings, by contrast, can extend from 100 nm up to 2 µm in length.

Under control conditions, microtubules were damaged, in general, with a frequency of 0.14 events per micrometer (Fig. 2c), comparable to previous reports^24,58^. Among these, 43% were kinks, 43% dimer dissociation, and 14% were sheet openings along the lattice (Fig. 2e). Note that microtubule damage has previously been reported using various techniques^30,59–61^ but could be amplified by the staining procedure on the EM grid (Fig. 2b, and Supplementary Fig. 2a; see “Methods”).

Snap freezing significantly increased the incidence and severity of lattice damage. Microtubules were affected, with a frequency of 0.5 events per micrometer (Fig. 2c). Notably, sheet openings became more extensive, reaching up to 2 µm in length, and their occurrence rose by 17-fold, compared to controls (Fig. 2d). Upon snap freezing, all four damage types were observed, including broken microtubules and long sheet openings (Fig. 2d–f, and Supplementary Fig. 2a).

Kinesin-1Δ6 treatment at a 1:2 kinesin:tubulin ratio also induced substantial microtubule damage with a frequency of 0.54 events per micrometer (Fig. 2c); however, unlike snap freezing, the predominant damage was breaks, accounting for 55% of all observed events - indicating a qualitatively distinct damage profile (Fig. 2e, f). At a lower kinesin-1Δ6 concentration (1:24 ratio), the motor primarily induced kink-type damage, which made up 59% of the total damage (Fig. 2e, f, and Supplementary Fig. 2a). This suggests that at higher concentrations, kinesin-1Δ6 breaks microtubules by first generating lattice kinks that eventually lead to rupture (Fig. 2e, f, and Supplementary Fig. 2a). Sheet opening, however, only increases 1.5-fold upon motor running on microtubules, which were also shorter, ranging between 100 and 900 nm, compared to snap freezing induced openings (Fig. 2d). Together, these findings establish two distinct approaches that produce robust yet qualitatively different forms of microtubule lattice damage: snap freezing, which favors extensive sheet openings, and kinesin-1Δ6, which predominantly generates kinks, breakage and shorter sheet openings.

### Microtubule damage reduces αTAT1 acetylation activity and increases HDAC6 deacetylation

**Fig. 3:**
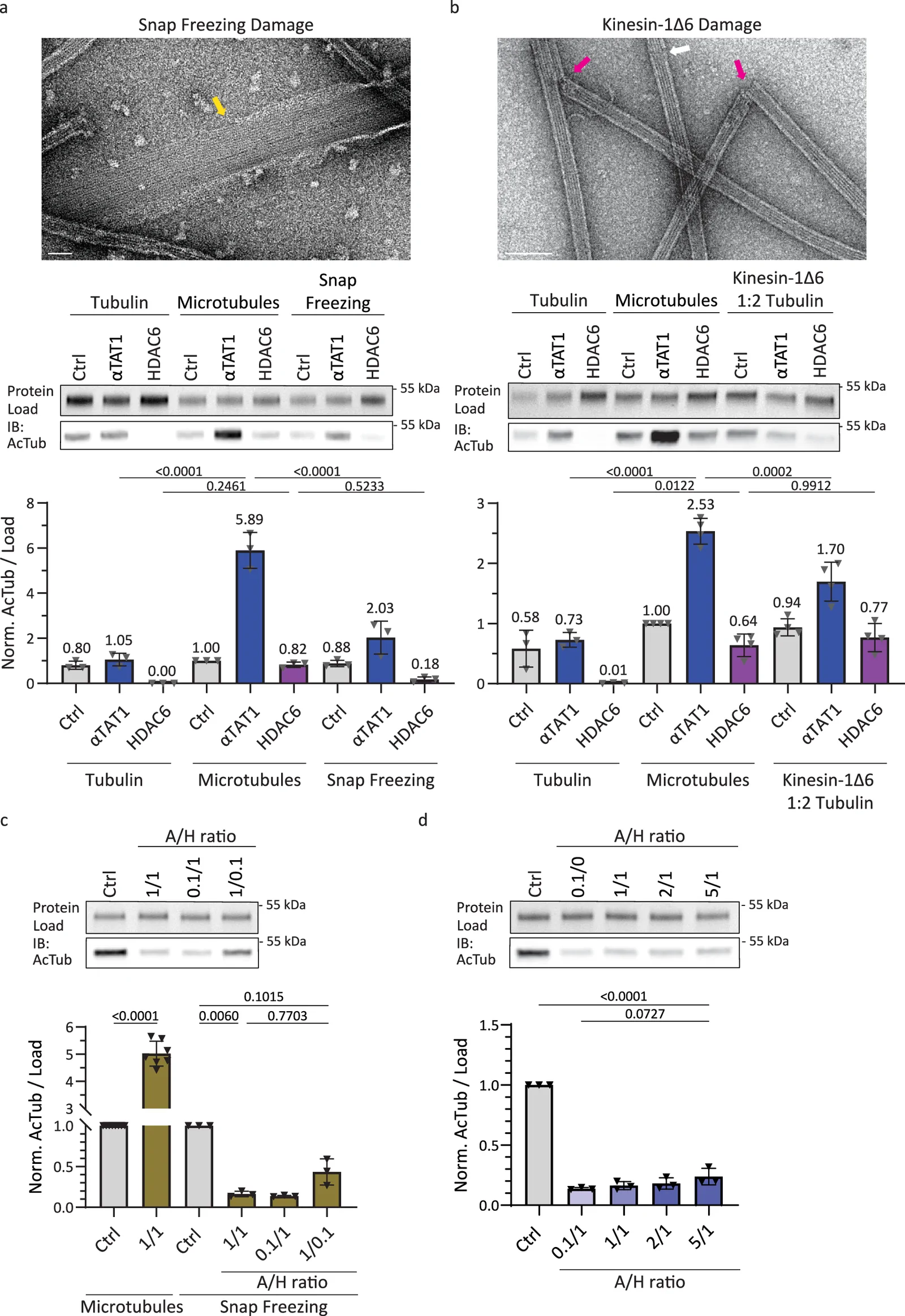
Microtubule damage reduces αTAT1 acetylation efficiency. Representative EM images of GMPCPP-microtubules (top) after **a** snap freezing or **b** kinesin-1Δ6-induced damage. Damage types are indicated with color-coded arrows: yellow – sheet opening, magenta -kink, white dimer dissociation. Scale bars: 100 nm. Representative Western blot analysis (middle) and quantification (bottom) of acetylation levels all for αTAT1 (1 µM), HDAC6 (1 µM) or no enzymes (Ctrl) for both damage conditions, in comparison to tubulin and intact microtubules. AcTub levels are normalized to protein load. Statistics: one-way ANOVA. Mean with SD. **c**, **d** Representative Western blot analysis for (de)acetylation reactions for snap frozen GMPCPP-microtubules (1 µM). **c** Competition experiment using different A/H ratios (αTAT1/HDAC6, enzymes 0.1 or 1 µM) with damaged compared to intact microtubules from Fig. 1c and **d** Reactions with increasing αTAT1 concentrations at constant HDAC6. **a**–**d** Quantifications of AcTub levels relative to the protein load, from three or four independent experiments. Statistics: one-way ANOVA. Mean with SD.

We next tested how damage in general - and how these different damage types in particular - affect the enzymatic efficiency of αTAT1 or HDAC6. HDAC6 efficiency to deacetylate kinesin-1Δ6 damaged microtubules is comparable to intact microtubules (Fig. 3b). In contrast, HDAC6 showed markedly higher activity on snap-frozen microtubules, resulting in an 80% reduction of brain tubulin acetylation levels. This is much closer to the enzyme’s efficiency on free tubulin and is significantly greater than the 20% reduction observed in undamaged microtubules (Fig. 3a). These results suggest that the large sheet openings caused by snap freezing enhance HDAC6 access to the luminal K40 site (Supplementary Fig. 2c).

Due to its small size and rapid diffusion, αTAT1 equilibrates between the microtubule lumen and the cytosol within minutes in a 10-μm-long microtubule^48^. Detectable gradients or acetylation patches near damage would require microtubules longer than ~150 μm^48^. Acetylation at K40 is therefore limited not by access to the lumen, but by the inherently slow catalytic rate of αTAT1 (*k*_cat_ = 21.29 × 10^−4 ^s^−1^)^62^. While αTAT1 can enter through damage sites, it does not rely on them to access K40^52^; entry through microtubule ends is sufficient.

Although microtubule damage can, in principle, grant luminal access, we found that microtubule damage does not enhance αTAT1’s effective activity - in fact, it impairs it. Kinesin-1Δ6-treated microtubules showed a twofold reduction in αTAT1-mediated acetylation compared to undamaged controls (Fig. 3b). This effect was further amplified by snap freezing, where damaged microtubules showed a fivefold reduction in acetylation, which accounts for only a twofold increase in acetylation, compared to a nearly sixfold increase in intact microtubules (Fig. 3a). This unexpected decrease in αTAT1 efficiency correlates with the degree of lattice disruption. One possibility is that damaged sites allow αTAT1 to exit the microtubule lumen before efficient acetylation of K40 can occur, thereby reducing overall acetylation levels in damaged microtubules. However, the reduction in acetylation does not scale with damage frequency - an increase of 0.3 events per micrometer is unlikely to explain a twofold reduction in acetylation. This suggests that damaged sites exert long-range effects on the microtubule lattice that impair acetylation efficiency beyond the immediate vicinity of the defect. Given that αTAT1 binding depends on lateral tubulin interactions^48^, we propose that αTAT1 requires an intact and continuous microtubule lattice for effective recognition of the K40 loop and subsequent acetylation (Supplementary Fig. 2c).

### HDAC6 outweighs αTAT1 activity on damaged microtubules

To directly test how enhanced luminal access affects the balance between acetylation and deacetylation, we next performed competition experiments on snap-frozen microtubules (Fig. 3c, d). In contrast to intact microtubules, where equimolar αTAT1 and HDAC6 resulted in a ~5-fold increase in acetylation (Fig. 1c), snap-frozen microtubules showed a net ~6-fold reduction in acetylation relative to untreated microtubules. This corresponds to an approximately 30-fold difference in acetylation levels between intact and damaged microtubules and indicates that HDAC6 activity dominates in damage conditions. Reducing the HDAC6 concentration tenfold while keeping αTAT1 constant partially alleviated this effect, resulting in a reduced but ~2-fold increase in acetylation (Fig. 3c).

Consistent with these findings, increasing αTAT1 concentration up to 50-fold at constant HDAC6 level did not measurably increase acetylation on snap-frozen microtubules (Fig. 3d). Together, these data show that αTAT1-mediated acetylation is effectively low on snap-frozen microtubules in the presence of HDAC6. Although αTAT1 retains some capacity to acetylate snap-frozen microtubules — albeit at reduced efficiency compared to intact lattices (Figs. 1b, c and 3c, d) — its activity is outweighed by HDAC6-mediated deacetylation (Fig. 3c, d). Given the markedly higher catalytic efficiency (40,000-fold *k*_cat_/K_M_) of HDAC6 toward free tubulin^54^ compared to αTAT1 acting on polymerized microtubules^62^, increased access to the microtubule lumen generated by snap freezing likely shifts HDAC6 activity toward a regime closer to that observed for free tubulin. Consequently, even a 50-fold excess of αTAT1 is insufficient to counterbalance HDAC6 activity in damaged microtubules.

### αTAT1 efficiently acetylates expanded microtubule lattices

Microtubule structural states play a role in modulating interactions with MAPs - which can read out and/or induce lattice compaction or expansion—including EB3, DCX^9,10^, TPX2^11^, MAP7 and tau^12,13^. Likewise, PTM levels at the microtubule surface, such as tubulin detyrosination, are influenced by the conformational state of the lattice^63^. We therefore asked whether αTAT1’s acetylation efficiency is influenced by specific microtubule lattice conformations.

**Fig. 4:**
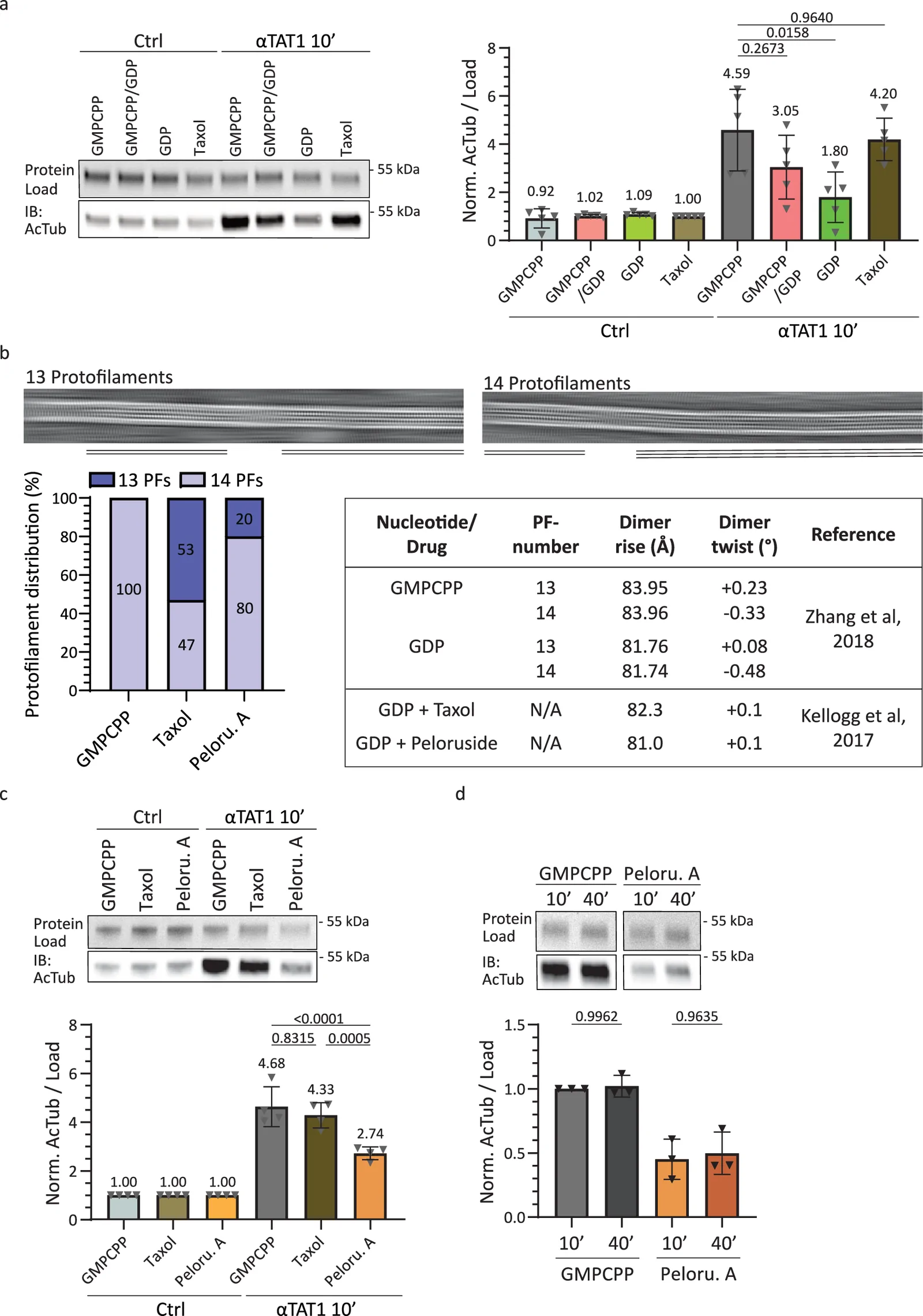
Lattice expansion enhances microtubule acetylation. **a** Representative Western blot analyses and quantification for microtubule acetylation after 10 min incubation with αTAT1. Microtubules were stabilized with GMPCPP, GMPCPP/GDP (1:1), GDP, or taxol and compared to no-enzyme (Ctrl). GDP microtubules result from GTP hydrolysis shortly after incorporation at the growing microtubule end. AcTub levels are normalized to protein load. **b** Representative filtered EM images show moiré patterns for 13-PF (2/0/2) and 14-PF (2/0/3) (see “Methods” for more details). Protofilament distribution (%) for GMPCPP- (*n* = 15), taxol- (*n* = 15) or peloruside A- (*n* = 15) stabilized microtubules. Reported PF number, dimer rise, and lattice twist values for the respective nucleotide/drug conditions are indicated according to Zhang et al.^8^ and Kellogg et al.^5^ **c**, **d** Representative Western blot analyses for acetylation reactions with αTAT1 on GMPCPP-, taxol-, or peloruside A- stabilized microtubules. **c** Acetylation after 10 min incubation compared to control conditions (Ctrl, no enzyme). **d** Acetylation after 10 or 40 min incubation. Quantifications show AcTub levels normalized to protein load from five (**a**), four (**c**), or three (**d**) independent experiments. Data represent mean ± SD; one-way ANOVA.

To fine-tune lattice spacing, we polymerized microtubules under different conditions: in the presence of GMPCPP, GTP, a 1:1 mixture of GMPCPP and GTP, or taxol. Here, to ensure nucleation across all conditions, microtubules were nucleated using GMPCPP- and taxol-stabilized seeds. In the presence of GMPCPP or taxol, microtubules are known to adopt an expanded lattice state (84 Å and 82.3 Å respectively)^4^. The 50:50 GMPCPP/GDP condition was designed to generate an intermediate lattice state, while GDP alone (after GTP hydrolysis) yields a compacted lattice (81.5 Å)^4^. When incubated with αTAT1 for 10 min, GMPCPP- and taxol-stabilized microtubules showed the highest acetylation levels, with a 4.59-fold and 4.20-fold increase, respectively (Fig. 4a). The intermediate GMPCPP/GDP state showed less increase in acetylation with 3.05-fold, and a GDP-compacted microtubule lattice led to the lowest acetylation increase of only 1.80-fold.

We expected that the microtubule lattice expands near damage sites, which - based on our findings with GMPCPP- and taxol-stabilized microtubules - should enhance acetylation by αTAT1. However, our results with kinesin-1Δ6–treated and snap-frozen microtubules contradict this assumption: instead of increased acetylation, we observed a reduction. One possible explanation is that tubulin does not expand near damage sites. Another one is that local, uneven protofilament expansion around damage sites disrupts lateral tubulin contacts, impairing αTAT1’s ability to recognize and modify the K40 loop. Unfortunately, we currently lack tools to resolve the tubulin and loop structure around a damage site at high resolution, so we relied on indirect, lattice-modulating assays as further readouts.

In addition to lattice compaction and expansion, different nucleotides and MAPs can alter the twist of tubulin dimers within the microtubule lattice, which can be reflected by the protofilament number^8,64,65^. To distinguish the effects of lattice spacing from dimer twist, we tested another stabilizing drug: peloruside A. We observed that all GMPCPP-stabilized microtubules adopted a 14-PF conformation, whereas peloruside A treatment resulted in predominantly 14-PF microtubules (80%). In contrast, taxol-stabilized microtubules displayed a more heterogeneous distribution, with 47% 14-PF and 53% 13-PF lattices (Fig. 4b).

Unlike GMPCPP and taxol, peloruside A locks microtubules into a compacted lattice conformation (81.0 Å)^5^ when added during polymerization and induces a positive lattice twist of ~0.1°^5^. Taxol, while expanding the lattice, induces a similar twist (~0.1°)^5^, whereas GMPCPP-stabilized microtubules with 14-PFs exhibit the highest negative twist (~-0.33°). We therefore compared αTAT1 activity on microtubules stabilized with GMPCPP, taxol, or peloruside A (Fig. 4c, d).

After 10 min of incubation, peloruside A-treated microtubules showed a 2.74-fold increase in acetylation (Fig. 4c) - higher than GDP-microtubules, despite both conditions sharing a similar compacted lattice but differing in lattice twists (Fig. 4b). However, peloruside A-stabilized microtubules were significantly less acetylated than GMPCPP-stabilized microtubules with the same PF number, or taxol-stabilized ones, which showed a 4.33-fold increase despite having a comparable twist but a more expanded lattice (Fig. 4b, c). Extending the incubation time to 40 min did not substantially increase acetylation levels in peloruside A-treated microtubules, indicating that the observed differences in αTAT1 efficiency are not due to delayed kinetics but instead reflect persistent restrictions on catalytic activity (Fig. 4d). Taken together, these results show that while increased lattice twist may modestly enhance αTAT1 activity, lattice expansion has a greater impact on its enzymatic efficiency.

## Discussion

Microtubule shaft damage can arise from both mechanical and physiological cues. In our reconstituted system, we artificially introduce mechanical damage through repeated freezing and thawing cycles of microtubules (snap freezing). We mimic physiological damage by using a highly damaging mutant of kinesin-1 (kinesin-1Δ6) to reinforce motor protein-induced damage. In both cases, the stress exerted on microtubules leads to tubulin dimer dissociation from the lattice, leaving behind a void within the polymer (Fig. 2). Motor stepping along the microtubule shaft most likely prompts local dissociation of multiple dimers within and across protofilaments, which results in the appearance of “kink” formations along the microtubule. Enhanced motor activity for high kinesin-1Δ6 concentrations promotes locally increased dimer dissociation, resulting in breakage of the tube, referred to as “break” (Fig. 2b–f). As these experiments were performed using GMPCPP-stabilized microtubules and considering the reported micrometer length of damage and incorporation sites on GDP-microtubules^30–33^, we propose that motor activity on GDP-microtubules would produce more extended lattice damage.

The enzymes αTAT1 and HDAC6 both require access to the microtubule lumen for modification of the α-tubulin K40 residue. We and others showed that HDAC6 deacetylates non-polymerized tubulin dimers more efficiently than polymerized microtubules, most likely due to its size that limits its entry and diffusion in the microtubule lumen^54^ (Fig. 1b). The catalytic domain of HDAC6 comprises an area of 4 × 10 nm according to AlphaFold predictions (AF-Q9UBN7), which is marginally smaller than the 14 nm inner diameter of a microtubule^66^. Upon pronounced microtubule damage, we hypothesize that HDAC6 can reach the K40 loop more easily, and deacetylation therefore increases (Fig. 3a). While small damages encompassing only a few dimers seem to be not sufficient to grant access (Fig. 3b). These results are in line with previous findings on higher HDAC6 deacetylation efficiency on polymerized Zn sheets or “inside-out” tubulin-dolastatin rings compared to intact microtubules^54^. In cellular contexts, small lattice defects may be further enlarged by mechanical forces or protein interactions, thereby enabling HDAC6-mediated deacetylation.

αTAT1-mediated acetylation, on the other hand, increases more than fivefold for polymerized microtubules (Fig. 1b,c). The structured region of αTAT1 comprises an area of only 3 × 5 nm (PDB: 4GS4). Hence, the rather small enzyme can enter through microtubule ends and freely diffuse through the lumen to modify the K40 residue of a 10 μm microtubule within 8 min^48^. αTAT1 efficiency is therefore dictated by its slow enzymatic activity rather than its access to the microtubule lumen^48^. Nevertheless, shaft damage induced by snap freezing of microtubules or by incubating microtubules with kinesin-1Δ6 motor proteins drastically decreases enzymatic efficiency for αTAT1 by up to 65% (Fig. 3).

We propose that αTAT1 is sensitive to microtubule lattice integrity. Any kind of damage to the microtubule shaft may alter the conformation of tubulin dimers in the lattice near the damage site, interfering with αTAT1’s ability to recognize the K40 loop. Our results demonstrate that αTAT1 can detect microtubule conformation ranging from compacted (81 Å) to expanded (84 Å) lattice states (Fig. 4) and respond accordingly. Hereby, the detected changes in acetylation efficiency are independent from nucleotide or protofilament number. For example, GMPCPP- and taxol-stabilized microtubules - despite having 14 and 13 protofilaments, respectively - are acetylated to similar levels, indicating that protofilament number does not influence αTAT1 activity under these conditions. However, acetylation efficiency decreases by half when microtubules are in a compacted state compared to expanded lattices (Fig. 4a, c, d). To acetylate, αTAT1 needs to recognize the K40 loop with its active binding site to transfer the acetyl group from Acetyl-CoenzymeA (AcCoA) to the lysine40 of α-tubulin, whereby the lateral contact to neighboring microtubules is important^48,67^. We suggest that a compaction of tubulin dimers limits access to the K40 loop exposed to the microtubule lumen and therefore reduces the efficiency of αTAT1 acetylation.

Overall, our findings show that αTAT1 preferentially acetylates intact microtubules with an expanded lattice conformation. Although acetylation correlates with long-lived microtubules^48,68^, these microtubules are not uniformly acetylated but instead display discrete acetylated segments^52^. One possible explanation is intermittent deacetylation by HDAC6. Supporting this, we previously showed that increased kinesin-1 activity promotes HDAC6-dependent deacetylation, likely because motor-induced lattice damage—often extending over hundreds of nanometers—creates access points to the lumen^52^.

Since MAPs can modulate microtubule lattice spacing^8,11,12,16^, an additional explanation is that such regulation could influence where and how efficiently αTAT1 acts within cells. Although kinesin-1 can transiently expand the lattice^39,40^, this effect occurs over short timescales compared to acetylation and is unlikely to support substantial acetylation. In contrast, more stable MAP binding could locally promote lattice expansion and thereby enhance αTAT1 activity, similar to the elevated acetylation observed in GMPCPP-stabilized microtubules (Fig. 4). Consistent with this idea, MAP7 binding to the outside of the microtubule correlates with increased αTAT1 acetylation^52,69^. A comparable mechanism has been proposed for detyrosination, where lattice conformation regulates the activity of the VASH1/SVBP complex^63^. Together, these observations support a broader emerging model in which the conformational state of the microtubule lattice acts as a regulatory layer that determines enzymatic activity on microtubules. In this framework, acetylation may preferentially mark stable microtubules that locally adopt an expanded lattice state. Identifying factors that can sustain lattice expansion over sufficient timescales for αTAT1 activity will be an important direction for future work.

How these local conformational changes and local deacetylation together contribute to the spatial organization of acetylation patterns - such as the acetylation gradients observed with its distinct acetylation segments along microtubules in cells - remains unsolved. In cells, acetylation distributes in a gradient with high perinuclear acetylation that decreases towards the cell periphery and running motor proteins modulate the gradient through microtubule damage^52^. Local damage caused by motor proteins could be a general mechanism to control lattice conformation of a subset of microtubules, together with MAPs regulating the lattice spacing. Tubulin-modifying enzymes like αTAT1 and the VASH1/SVBP complex are sensitive to such structural changes of the microtubule shaft, leading to spatially patterned PTMs of the microtubule network in cells.

## Methods

### Plasmid generation

From pEF5B-FRT-GFP-αTAT1(D157N) (addgene #27100), the amino acid at position 157 was mutated to aspartate, and the GFP sequence was replaced by a Flag-tag using AgeI/BglII restriction enzymes. Flag-αTAT1 was subcloned into a pET29b vector for bacterial protein expression using KpnI/BamHI restriction enzymes. The highly damaging constitutively active kinesin-1 mutant vector (kinesin-1Δ6-GFP) was generated as previously described^52^ and cloned into pET17-K560-GFP-His (addgene #15219) using EcoRI/AgeI restriction enzymes. The HDAC6 sequence from HDAC6-Flag (addgene #13823) was subcloned into a pFastBachHTA vector using PluTI/NotI restriction enzymes for Baculovirus generation and subsequent Sf9 cell infection.

### Tubulin purification and labeling

Tubulin was purified from bovine brain by two polymerization/depolymerization cycles in High-Molarity PIPES buffer as described previously^70^. In brief, a first polymerization/depolymerization was performed in High-Molarity PIPES buffer (1 M PIPES, pH 6.9, 10 mM MgCl_2_, 20 mM EGTA, 1.5 mM ATP, 0.5 mM GTP) supplemented with 1:1 glycerol and Depolymerization buffer (50 mM MES at pH 6.6, 1 mM CaCl_2_), respectively. A second polymerization/depolymerization was performed with polymerization in High-Molarity PIPES buffer and depolymerization in 0.25× BRRB80 with addition of 5× BRB80 after 15 min to reach 1xBRB80 (80 mM PIPES at pH 6.8, 1 mM MgCl_2_, 1 mM EGTA).

Purified tubulin was labeled with ATTO-488, ATTO-565, ATTO-647 (ATTO-TEC GmbH) or biotinylated tubulin, adapted from a previously published protocol^71^. Briefly, tubulin was polymerized in glycerol PB solution (80 mM PIPES pH 6.8, 5 mM MgCl2, 1 mM EGTA, 1 mM GTP, 33% glycerol) for 30 min at 37 °C and placed in layers onto cushions of 0.1 M NaHEPES pH 8.6, 1 mM MgCl_2_, 1 mM EGTA, and 60% glycerol and centrifuged. The pellet was resuspended in resuspension buffer (0.1 M NaHEPES pH 8.6, 1 mM MgCl_2_, 1 mM EGTA and 40% glycerol), followed by incubation for 10 min at 37 °C with either 1/10 volume of 100 mM ATTO-488, -565, or -647-NHS-fluorochromes or for 20 min at 37 °C with 2 mM NHS-biotin. Labeled tubulin was sedimented onto cushions of 1× BRB80 supplemented with 60% glycerol, resuspended in BRB, and a second polymerization/depolymerization cycle was performed. The labeling ratio was 11% for ATTO-488 and 12% for ATTO-565.

### Protein expression and purification

For purification of *αTAT1*, *Escherichia coli* BL21 (DE3 pLysS) cells were transformed and grown at 37 °C until an optical density at 600 nm of 0.6–0.8 and induced for protein expression with 1 mM IPTG at 18 °C overnight. Cells were harvested and lysed in lysis buffer (50 mM Tris pH 7.5, 150 mM NaCl, 10% glycerol) supplemented with 1% Triton-X 100, 2 mM DTT, 2 mM PMSF and protein inhibitor cocktail (Roche, 11873580001) and subsequently sonicated. Lysed cells were cleared by centrifugation at 12,000 rpm for 30 min at 4 °C. Proteins were affinity purified using a 1 mL HisTrap HP column (GE Healthcare), washed in lysis buffer supplemented with 0.2% Triton-X 100 and 25 mM imidazole and eluted with 250 mM imidazole within 10 column volumes. Fractions were pooled and concentrated using Amicon 30K Centrifugal Filters (Millipore). Concentrated proteins were loaded onto a size-exclusion chromatography using a HiLoad 16/600 Superdex 200 column (GE Healthcare) in 20 mM Tris pH 7.5, 150 mM NaCl, 1 mM MgCl_2_. Collected fractions were concentrated, and concentration determined by Bradford assay. Proteins were supplemented with 10% glycerol, aliquoted, snap-frozen in liquid nitrogen and stored at −80 °C.

For *kinesin-1*Δ*6-GFP* (referred to as: kinesin-1Δ6) a similar protein purification protocol was used. Protein expression was induced with 0.5 mM IPTG at 16 °C overnight. Cells were lysed in 100 mM Na_2_HPO_4_/NaH_2_PO_4_ pH 7.5, 600 mM KCl, 3 mM MgCl_2_, 10% glycerol, 20 mM β-mercaptoethanol, supplemented with 10 mM imidazole, protein inhibitor cocktail and 100 mM ATP addition right before lysis. Cell lysates were ultracentrifuged at 40,000 rpm for 50 min at 4 °C. Proteins were loaded on a 1 mL HisTrap HP column, washed with 30 mM imidazole, washed again with 60 mM and eluted with a 60–500 mM imidazole gradient over 30 column volumes. Size-exclusion chromatography was performed in 10 mM HEPES pH 7.4, 150 mM KCl, 1 mM MgCl_2_, 0.05 mM ATP, 1 mM DTT and concentrated proteins were supplemented with 20% sucrose before snap-freezing and storage at −80 °C.

*HDAC6* was purified from insect cells by resuspending the cell pellet in lysis buffer (100 mM Tris pH 8, 10 mM NaCl, 5 mM KCl, 2 mM MgCl_2_, 10% glycerol, 0.2% Triton-X) supplemented with protein cocktail inhibitor. After lysis, NaCl concentration was adjusted to 150 mM, and cells were ultracentrifuged at 50,000 rpm for 20 min at 4 °C. Affinity chromatography was performed using a 1 mL HisTrap HP column and proteins were washed with 25 mM imidazole and eluted from the column with 250 mM imidazole within 5 column volumes. Concentrated proteins were further purified by size-exclusion chromatography in lysis buffer and concentrated proteins were supplemented with 10% glycerol, snap-frozen and stored at −80 °C.

### Microtubule seed preparation

Short microtubule seeds were prepared by mixing 30% ATTO-647-labeled tubulin with 70% biotinylated tubulin with a final concentration of 10 μM in 1× BRB80. The solution was supplemented with 0.5 mM GMPCPP and incubated for 45 min at 37 °C. After the addition of 1 μM Paclitaxel (taxol), the solution was incubated again for 30 min at 37 °C and subsequently centrifuged for 15 min at 14,000 rpm at room temperature (RT). The microtubule pellet was resuspended in 1× BRB80 supplemented with 0.5 mM GMPCPP and 1 μM taxol. Seeds were aliquoted and stored in liquid nitrogen.

### Microtubule assembly

GMPCPP-stabilized microtubules were polymerized by incubating 5 μM ATTO-488 or ATTO-565 pre-labeled tubulin mix in 1× BRB80 buffer (80 mM PIPES pH 6.8, 1 mM MgCl_2_, 1 mM EGTA) supplemented with 0.5 mM GMPCPP for 20 min at 37 °C. To increase the length, two subsequent additions of 1 μM tubulin for 10 min at 37 °C were performed. The tubulin mixture was centrifuged at 12,000 rpm for 15 min at RT, and microtubules were resuspended in 1× BRB80. For comparison with other stabilized microtubule lattices (Fig. 4), GMPCPP microtubules were assembled from 10 μM directly without sequential tubulin addition.

### Microtubule (de)acetylation

GMPCPP-stabilized microtubules (corresponding to 1 μM tubulin in the final volume) were diluted in 1×BRB supplemented with 100 μM Acetyl CoenzymeA (Sigma-Aldrich, A2056) and 1 mM DTT. For (de)acetylation reactions with equimolar ratios, 1 μM αTAT1, 1 μM HDAC6, or the same volume of HDAC6 purification buffer as a control were added to different tubes, respectively. For enzyme competition experiments, 1 μM αTAT1 and 1 μM HDAC6 were added to the same tube, or either αTAT1 or HDAC6 concentrations were lowered by tenfold to 0.1 μM. As a control for microtubule acetylation, the same procedure was performed with non-polymerized tubulin (1 μM). Reactions were incubated for 1 h at RT, and samples taken for Western blotting or immunostaining from the same sample (for microtubules only).

### Microtubule acetylation assay for different lattices

For generating microtubules with different microtubule lattices, experiments in Fig. 4a, 10 μM ATTO-488 or ATTO-565 pre-labeled tubulin mix was incubated with 0.2 μM microtubule seeds (final concentrations in the reaction mix: 20 nM taxol, 10 μM GMPCPP) in 1× BRB80 buffer supplemented with 1 mM GMPCPP, 0.5 mM GMPCPP/0.5 mM GTP, 1 mM GTP or 1 mM GTP/10 μM taxol, respectively. Seeds were needed in these experiments to overcome the differences in nucleation rates between these conditions.

Taxol-, or peloruside A-stabilized microtubules were assembled similarly to GMPCPP-stabilized microtubules from 10 μM ATTO-488 or ATTO-565 pre-labeled tubulin in 1× BRB80 supplemented with 1 mM GTP and 1 μM taxol or 1 μM peloruside A (a kind gift from Michel Steinmetz, Peter Northcote and John Miller), respectively. Microtubules were polymerized for 30 min at 37 °C and 10 μM αTAT1 with 100 μM Acetyl Coenzyme A was subsequently added to each mixture and incubated for 10 min at 37 °C. Reactions were centrifuged at 12,000 rpm for 15 min at RT, and microtubules were resuspended in 1× BRB. Samples were taken for Western blotting or immunostaining.

### Microtubule damage generation

GMPCPP-stabilized microtubules (7 μM tubulin) were exposed to five subsequent cycles of snap freezing in liquid nitrogen for 1 min, followed by thawing the solution back to RT for 1 min. After completion of freezing/thawing cycles, microtubules were immediately stained for electron microscopy or used for (de)acetylation assays. For snap freezing control conditions, untreated GMPCPP-microtubules were used.

For damaging microtubules with kinesin-1Δ6, GMPCPP-stabilized microtubules (3.5 μM tubulin) were incubated with either 1.75 μM or 84 nM of kinesin-1Δ6 for a 1:2 or 1:24 tubulin ratio respectively, in 1× BRB supplemented with 2.7 mM ATP. Control conditions were performed in 1× BRB with 2.7 mM ATP. Reactions and Control were incubated for 15 min at 37  °C and immediately stained for electron microscopy or used for (de)acetylation assays.

### Negative stain transmission electron microscopy

For negative stain transmission electron microscopy (negative stain EM), damaged microtubules were diluted in 1× BRB to a final concentration of 3.5 µM. A 4 µL drop of the sample was applied directly onto glow-discharged copper EM grids (Electron Microscopy Sciences). After a 1-min incubation, the excess sample was blotted off, and the grids were washed twice with 10 µL drops of 2% uranyl acetate (Electron Microscopy Sciences), each time followed by immediate blotting. The grids were then stained with a 10 µL drop of 2% uranyl acetate for 1 min, blotted, and air-dried for 2 min. Prepared grids were stored in EM grid boxes at RT until imaging. Imaging was performed using a Talos L120C transmission electron microscope.

### Microtubule damage characterization

Microtubule damage was classified into four categories: dimer dissociation, kink, break, and sheet opening.

Image analysis of electron microscopy images was performed in ImageJ and restricted to single microtubules. Microtubule bundles were excluded from all analyzes. To quantify damage *frequency* (event/µm), the length of each microtubule was measured, and total microtubule length was summed across all fields of view for each condition. Individual damage events (for each damage type separately) were counted and expressed as events per micrometer by dividing the total number of events by the corresponding total microtubule length. The *fraction* of microtubules in an open-sheet configuration was calculated for each condition by dividing the summed length of lattices' open-sheet configuration by the total microtubule length.

Dimer dissociation was defined as the absence of protein density corresponding to at least one tubulin dimer along a protofilament, or as outward curling of a protofilament within the lattice. A kink was defined as a sharply bent microtubule with visible lattice disruption at the bending site.

Breaks were defined as two proximal microtubule ends consistent with lattice rupture. As negative-stain EM cannot distinguish true breaks from two closely adjacent independent microtubules, only ends observed within <100 nm of each other were scored as breaks. This criterion was applied uniformly across all conditions, including controls. Note that this criterion might overestimate the occurrences of breaks. Sheet opening was defined as the unfolding of the microtubule tube into an open sheet structure. For quantification of sheet opening, the cumulative length of opened lattice regions was divided by the total microtubule length measured, yielding the fraction of lattice in an open-sheet configuration for each condition. In snap-frozen samples, sheet opening frequently resulted in ribbon-like structures. Folding of these sheets limited reliable length measurements; therefore, sheet-opening lengths in these samples likely represent an underestimate.

### Microtubule (de)acetylation of damaged microtubules

Intact, snap freezing- or kinesin-1Δ6-damaged microtubules (concentration in final mix: 1 μM tubulin) or non-polymerized tubulin (1 μM) were diluted in 1×BRB supplemented with 100 μM Acetyl CoenzymeA and 1 mM DTT. For (de)acetylation reactions, 1 μM αTAT1 or 1 μM HDAC6 or the same volume of HDAC6 purification buffer as a control was added, respectively. For enzyme competition experiments, αTAT1 or HDAC6, respectively, were added stable at 1 μM, while the other enzyme was added to the same tube at either 0.1, 1, 2, or 5 µM for αTAT1 or 0.1 or 1 µM for HDAC6. Reactions were incubated for 1 h at RT and samples taken for Western blotting.

### Microtubule protofilament analysis from TEM

To determine protofilament number, microtubules were analyzed from negative-stain TEM micrographs. Straight microtubule segments of approximately 500 nm were selected based on uniform contrast and minimal curvature. Segments containing visible lattice defects, ends, or abrupt changes in contrast were excluded from analysis. All image processing and measurements were performed in ImageJ.

Protofilament number was determined from the same filtered microtubule segments using moiré pattern analysis, following established methods^14,72^. For each segment, a mask was generated from the midplane signal line and the 1/4-nm layer line in the Fourier transform. The mask was applied to the complex FFT, followed by an inverse FFT to recover the filtered image. The resulting moiré patterns were then inspected and scored to assign a protofilament number. Moiré nodes were defined as positions along the microtubule where the long-range interference envelope vanished, corresponding to phase inversions in the projected lattice. Intensity line profiles were extracted immediately before and after each node, and the number of repeating lattice density maxima was counted on either side. Only microtubules displaying clear, regular moiré modulation were included in the analysis.

Characteristic node-centered patterns were observed: a 2/0/2 pattern corresponded to 13 protofilaments, a 2/0/3 pattern to 14 protofilaments, and a 3/0/3 pattern to 15 protofilaments, consistent with established geometrical models of microtubule lattice interference^14^.

### SDS-PAGE and Western blot

Samples were diluted in 1× sample buffer (60 mM Tris, 2% SDS, 10% glycerol, 5% β-mercaptoethanol, 0.005% bromphenol blue) and run on 4-20% Mini-PROTEAN® TGX Stain-Free™ Protein Gels (BIO-RAD) in 1× running buffer (25 mM Tris, 192 mM Glycine, 0.1% SDS, pH 8.3). Protein load was detected by UV exposure of gels with a Fusion FX Vilber (Witec AG). Proteins were transferred to a nitrocellulose (NC) membrane of iBlot™ 3 Transfer Stacks (ThermoFisher Scientific) with an iBlot™ 3 Western Blot Transfer Device (ThermoFisher Scientific). NC membranes were blocked for 1 h with blocking buffer (5% dried milk in TBS-Tween 0.5%) and incubated with the primary antibody anti-acetylated tubulin (Sigma-Aldrich, T74451, 1:1000 dilution) in blocking buffer at 4 °C overnight. The next day, membranes were washed 3× with TBS-Tween 0.5% (TBS-T) and incubated for 1 h at RT with the secondary antibody anti-mouse-horseradish peroxidase (HRP) (GE Healthcare, NA9310, 1:5000 dilution) in blocking buffer. Membranes were washed again 3× with TBS-T, and HRP-conjugated proteins were revealed with a WesternBright® ECL detection kit (Advansta) on a Fusion Solo Vilber Lourmat camera (Witec AG). The UV protein load signal was used as a loading control for normalization of the anti-acetylated tubulin signal. The values for the acetylated tubulin antibody signal/UV protein load were normalized to a control condition in each experiment.

### Immunofluorescence

Coverslips were cleaned in two steps: sonication in 1 M NaOH for 40 min, rinsing in bidistilled water, followed by sonication in 96% ethanol for 40 min, repeated rinsing in bidistilled water and then dried with an air gun. In vitro polymerized microtubules were sedimented for 5 min on cleaned coverslips and subsequently fixed with 100% cold methanol for 4 min at −20 °C, followed by 3× washes with phosphate-buffered saline (PBS). Microtubules were permeabilized and blocked with blocking buffer [2% bovine serum albumin (BSA) and 0.1% Triton X-100 in PBS] for 5 min and subsequently incubated with the primary antibodies anti-acetylated tubulin (Sigma-Aldrich, T74451, mouse, 1:1000 dilution) or anti-α-tubulin (Geneva Antibody Facility, AA345, rabbit, 1:1000 dilution) in blocking buffer in a closed humidified chamber at RT for 15 min. Microtubules were washed 3× with blocking buffer and incubated with the secondary antibodies (Invitrogen, species-specific IgG conjugated to Alexa-647, or 488 fluorophores, 1:1000 dilution in blocking solution) for another 15 min in a closed humidified chamber at RT. Microtubules were washed again 3× with blocking buffer, and coverslips were mounted onto glass microscopy slides (Glass technology) and sealed with nail polish. Immunofluorescence images were taken using an Axio Observer Inverted TIRF microscope (Zeiss, 3i) and a Prime 95B (Photometrics). A 100X objective (Zeiss, Plan-Apochromat 100X/1.46 oil DIC (UV) VIS-IR). SlideBook 6×64 software (version 6.0.4) was used for image acquisition. As microtubules are fixed prior to antibody staining, some deformation or clumping is unavoidable, as well as background related to antibody on the glass.

### Image and statistical analysis

Immunofluorescence images were analyzed using ImageJ. For data analysis, the camera background of 100 a.u. was subtracted for each channel.

The *average acetylation level* of microtubules was quantified by measuring the fluorescence intensity profile of the acetylated tubulin antibody relative to the fluorescence intensity profile of the microtubule (ATTO-488- or ATTO-565-labeled tubulin as described above) and normalized to the control condition. For the analysis of *acetylation profile* along different subsets of microtubules, the ratio of the intensity values for acetylated tubulin/ATTO-tubulin was Ln (Natural Log) transformed and plotted over the normalized distance. To normalize microtubule length, the length of each line plot was divided by the respective maximum value. For comparison of individual microtubules, the normalized distance values were binned into 0.005 steps, and the combined acetylation profiles averaged. Unless otherwise stated, analyzes were restricted to single microtubules, and bundled microtubules were excluded or analyzed separately.

### Statistics and reproducibility

Statistical analyzes were performed by one-way ANOVA with Tukey’s multiple comparisons test using GraphPad Prism software (v.10.4.1). *P* values < 0.05 were considered statistically significant. Sample sizes were not predetermined using statistical methods and are indicated in the corresponding figure legends. All experiments were performed at least in triplicate with consistent results; replicates refer to independent experimental repeats. Control and experimental conditions were processed in parallel, and microtubules were randomly selected during image acquisition.

### **Limitations of the study**

A limitation in the study is the widely used, commercially available antibody for tubulin acetylation 6-11B-1 (Sigma-Aldrich, T74451) for in vitro assays, as previously reported. As described, the epitope recognition is perturbed in the presence of the microtubule stabilizing agent taxol^45^ and likely by other factors impacting the lattice structure (Fig. S1 Consequently, imaging analysis of immunofluorescence).

Breaks were defined as two proximal microtubule ends consistent with lattice rupture. Because negative-stain EM cannot unambiguously distinguish true breakage from two closely adjacent independent microtubules, only ends separated by <100 nm were scored as breaks. Although this criterion was applied uniformly across all conditions, it may lead to an overestimation of break frequency.

### Reporting summary

Further information on research design is available in the Nature Portfolio Reporting Summary linked to this article.

## Supplementary information

Transparent Peer Review file

Supplementary Information

Description of Additional Supplementary Files

Supplementary Data

Reporting summary

## Data Availability

All data associated with this study are presented in the manuscript in main figures, Supplementary Information and Supplementary Data. Source data including uncropped Western blots are provided in the Supplementary Information (Supplementary Figs. 3–5).
