# Supplementary material for: Microtubule lattice conformation and integrity regulate α-tubulin acetylation
Source: Commun Biol. 2026 Jun 8;9:1211. doi: 10.1038/s42003-026-10157-4 (PMC13582946; doi:10.1038/s42003-026-10157-4)
Supplement: Supplementary file 1 [file 42003_2026_10157_MOESM1_ESM.pdf]

nature portfolio

Peer Review File

Microtubule lattice conformation and integrity regulate α-tubulin acetylation

Corresponding Author: Professor Charlotte Aumeier

This file contains all reviewer reports in order by version, followed by all author rebuttals in order by version.

Version 0:

Reviewer comments:

Reviewer #1

(Remarks to the Author)
This paper addresses an important and currently intensely studied problem – the impact of microtubule lattice structure on post-translational tubulin modifications. The authors perform in vitro experiments with purified proteins to investigate the impact of microtubule damage and microtubule lattice conformations on acetylation of microtubules by alphaTAT and their deacetylation by HDAC6. The authors conclude that alphaTAT overrides the enzymatic activity of HDAC6, but is less active on strongly damaged microtubules. By comparing the acetylation efficiency of microtubules stabilized by different agents, the authors provide evidence that alphaTAT is sensitive to the expansion of the microtubule lattice.
If the data were of good quality, this study would have been an extremely useful addition to the field. Unfortunately, while the Western blots look good, the fluorescence microscopy and negative-stain TEM images are of poor quality, which makes the conclusions unreliable. The discussion of microtubule lattice conformation is superficial: it does not take into account that microtubules stabilized by different agents would have different protofilament numbers, which could strongly affect lattice twist. For this reason, the paper is not publishable in a peer-reviewed journal in its current form.

Specific comments:

1. Fluorescence images, for example in Fig. 1d, show many microtubule clumps; the patchy labeling for acetylated tubulin is not clearly visible. A comparison of the two channels — tubulin acetylation and total tubulin — along a single, well-resolved microtubule is needed for all conditions.

2. Imaging and quantifications of microtubule damage by negative-stain TEM are very unconvincing. For example, it is intrinsically impossible to distinguish “broken MTs” from two adjacent seeds unless one observes them live under a fluorescence microscope before and after breaking. This type of damage should be excluded from quantification. Dimer-removal examples are not convincing, and it is completely unclear how any damage can be quantified in microtubule bundles, which predominate in some conditions. If the images are not convincing, the corresponding quantifications and ensuing conclusions are not meaningful. Importantly, cryo-ET is currently the preferred method to characterize lattice defects. For any analysis, quantifying microtubules that are not overlapping or bundled with other microtubules is essential.

3. The discussion about lattice twist seems superficial and incorrect — GMPCPP-stabilized microtubule seeds in vitro typically have 14 protofilaments (and thus a negative twist). The authors should quantify the protofilament numbers of the different stabilized microtubule preparations they study and discuss their conformations taking these data into consideration.

Reviewer #2

(Remarks to the Author)
In this manuscript the dynamics of microtubule (MT) acetylation of lysine 40 of α-tubulin, a hallmark of stable MTs in cells, is investigated in vitro. The authors generate MTs with different properties (conformations and/or damage) and either add α-tubulin acetyltransferase 1 (ATAT1), the enzyme that generates this mark, or histone deacetylase 6 (HDAC6), which removes acetylation, or both enzymes. By modifying MT lattice conformation and introducing damage in defined manners the authors show that the acetylation efficiency of ATAT1, which overwrites HDAC6 enzymatic activity, decreases when MTs are damaged. They also find that ATAT1 efficiently acetylates MTs in expanded lattices and twisted tubulin states,

whereas compacted lattices reduce ATAT1 activity.

Given the cellular importance of lysine 40 acetylation of α-tubulin (in short: MT acetylation) the activities and functions of ATAT1 and HDAC6 have been examined by different groups. For example, the fact that ATAT1 prefers expanded MTs has been published (Ref 63). The main novelty of the present manuscript appears to be the finding that ATAT1 is sensitive to MT lattice integrity. I believe the results reported here are of importance for the field. I do have several comments, which I list below.

Comments.
1) In Fig. 1 ATAT1 and HDAC are added at defined concentrations, either alone or in combination. It would be interesting to use different enzyme concentrations. For example, is the roughly 5-fold increase observed with ATAT1 in Fig. 1b, c, a saturation effect or can this be further increased using higher concentrations of ATAT1? In Fig. 1c in the competition experiment a ratio of 0.1 ATAT1 to 1 HDAC6 is used. This leads to a 1.35-fold increase in acetylated tubulin. This experiment should be repeated with 0.1 ATAT1 alone, to see if there was any competition by HDCA6.

2) In Figures 2 and 3 visualization of MT damage, induced by snap freezing or mutant kinesin, by EM is shown. Four classes of damage are described by the authors, i.e dimer dissociation, kink, sheet opening and broken MTs. However, the "dimer removal" is very difficult to see in Fig. 2a and seems impossible to quantify accurately, also given the density of MTs in EM images (shown in Fig. 2d). Hence, the 14% dimer dissociation along the lattice (in Control) is likely an underestimation, and the other two values therefore an overestimation. Nevertheless, the qualitative effect of the damaging treatments seems clear. In Fig. 2e dimer removal is supposed to be indicated in blue and sheet opening in orange, however, the percentages associated with blue (43%) and orange (14%) in Control suggest the opposite, i.e. dimer removal is orange and sheet opening blue. If I am correct, the authors should correct this and carefully check their panels/labelling.

3) In Fig. 2b, c there is a condition called "No". I assume this means that no mutant kinesin was added, and that it is in essence a control. If so, I understand the same number of events per micron for "No" (0.15) and "Control" (0.14) in Fig. 2b. However, how can the values differ in Fig. 2c?

4) I do not understand the y-axis in Fig. 2c "Percentage of microtubule in sheet opening state observed along microtubules by EM for both damaging conditions". Could the authors explain this in more detail?

5) The experiments in Fig. 3 were done with either ATAT1 or HDAC6. Results in Fig. 3c suggest that large sheet openings caused by snap freezing enhance HDAC6 access to the luminal K40 site. Given the higher activity of HDAC6 it would be interesting to do a competition experiment as shown in Figure 1, also with higher concentrations of ATAT1.

6) Fig. 3c tries to visually explain the results obtained in Figure 3. However, while the more efficient entry of HDAC6 is represented in the panel, the reduction in enzymatic activity of ATAT1 due to damage, is not well seen. I would suggest to either remove this panel or to try and improve it.

7) On page 7, lines 201-204, the authors state "Notably, GMPCPP-microtubules - having the most expanded lattice - were slightly more acetylated than taxol-stabilized ones. The intermediate GMPCPP/GDP state showed less increase in acetylation with 3.05-fold, and a GDP-compacted microtubule lattice led to the lowest acetylation increase of only 1.80-fold". I am not a statistician but can such conclusions be drawn in the absence of statistical significance for some of the results (GMP vs Taxol: 0.9640, GMP vs GMP/GDP: 0.2673)?

8) Fig. 4b tries to visually explain MT conformations in the presence of different compounds, and their effects on acetylation. What is the value of panel 4b? Why present it before Fig. 4c?

9) In Fig. 4c the authors show a "representative" western blot (one of four experiments performed). However, in the "protein load" samples, the last two samples (Taxol, Peloruside A) have considerable less protein loaded. Since this is a representative result, how is this possible? How does this affect the quantification shown below the blot?

10) In the Methods section the authors mention that GMPCPP-stabilized seeds contain both Taxol (1 micromolar) and GMPCPP (0.5 mM). Since Taxol and and GMPCPP influence acetylation I wonder how these compounds are removed in subsequent experiments where seeds are used.

11) On page 9, lines 278-279, the authors state: "Acetylation could specifically mark stable microtubules that adopt an expanded lattice state". I thought that the long-lived nature of stable MTs explained increased acetylation. How did the authors reach the conclusion that stable MTs adopt an expanded lattice state?

Reviewer #3

(Remarks to the Author)
The manuscript by Egolt and colleagues investigates the mechanism by which acetylated tubulin is generated in microtubules by the enzymes aTAT and HDAC6. The authors show that enzymatic activity is sensitive to damage sites in the microtubule lattice, the expanded vs compacted state of the microtubule lattice, and the twist of the microtubule lattice. The work is important for the field but seems a bit preliminary as there are caveats and alternative explanations that could be addressed experimentally but should at a minimum be addressed in the text. The manuscript requires further text edits and image presentation to improve clarity and readability.

Major issues:

1) Figure 1 legend title “αTAT1 overwrites HDAC6 activity on intact microtubules in vitro“ and lines 106-107 “HDAC6 deacetylation activity is low in microtubules so that the presence of its counteractor αTAT1overwrites its activity.” This seems to be a mis-interpretation of the data. HDAC activity is low on microtubules (Figure 1b) so how can its counteractor aTAT “overwrite” it? Figure 1c basically shows that adding an equimolar amount of HDA6C (1:1 A:H ratio) or a bit of HDAC6 (1:0.1 A:H ratio) has no effect on AcTub levels. The only parameter that changes the amount of AcTub is to lower the amount of aTAT (0.1:1 A:H ratio). Thus, the proper interpretation would be that the presence of HDAC6 does not have any effect (positive or negative) on aTAT activity.

Figure 1d,e,f: since the AcTub antibody staining in this microscopy assay is inconsistent across samples (depending on what proteins are added to the assay), these data should be restricted to the supplement

Figure 1d seems to be low quality and thus difficult to visualize. In general, it is helpful to have microscopy images large and graphs small. Also, what are the boxes in the AcTub column for?

2) Figure 2 is difficult to parse with so many graphs and colors. The pie charts in 2e are particularly difficult to compare (a stacked bar graph may be better?) and basically show the same data as in 2f. Please think about how to better display this data. Perhaps separating the kinesin-1d6 and snap freezing data into different sections/graphs?

The criteria for each of these types of damage need to be clearly defined in the methods section. Particularly for dimer removal, this is not at all apparent from the image in 2a.

The types of damage under each condition in 2d should be marked with the same color arrows as shown in 2a.

How does the damage observed in these experiments compare to previous work using negative stain EM (Budaitis et al 2023, Reid et al 2017, Gonzalez et al 2024)?

3) For figure 3, an alternative possibility is that the damage sites permit aTAT to leave the microtubule lumen before it can generate AcTub and this is why there is less AcTub in the snap freeze and kinesin-1d6 damaged microtubules. At a minimum, this need to be discussed in the text.

4) The authors suggest that tubulin compaction vs expansion in the lattice regulates An alternative way to show that aTAT is sensitive to lattice expansion is to use the same microtubules (e.g .GTP or GMPCPP/GTP mix) and add a MAP that is known to expand the lattice such as MAP7 or the kinesin-1 rigor versus a MAP that is known to compact the lattice such as Tau or MAP2. Indeed, these methods were already used in the field to examine how expansion vs compaction influences tubulin detyrosination and the authors should mention this work (Yue et al 2023).

The authors also suggest that differences in microtubule twist are responsible for the differences in aTAT activity. I find this an interesting possibility but since this is the first report (to my knowledge) of how microtubule twist affects a microtubule modifying protein, the authors needs to be more thorough. What we know about microtubule twist is based on selected EM images and the twist of microtubules not included in these measurements is unknown. That is, we don’t really know the full extent of twist in the lattice under these different experimental conditions. The possibility that the effects are due to differences in protofilament number is mentioned in the Discussion but most microtubules are a mixture of protofilament numbers so unless the authors know the distribution of protofilament # microtubules across their conditions, this cannot be discounted.

Minor issues:

I am curious why the authors chose snap-freezing to generate damaged microtubules as opposed to exposing GMPCPP microtubules to CaCl2 (Gonzalez et al 2024) or generating open lattices using GTPgS (Reid et al 2017).

The manuscript text should state that the seeds are GMPCPP- and taxol-stabilized.

Lines 135-136: “as wildtype kinesin-1 damages stabilized microtubules insufficiently (see more details in Methods).” I could not find any additional details about this in the methods section.

Figure 4b needs a heading for each column.

Version 1:

Reviewer comments:

Reviewer #1

(Remarks to the Author)
The authors have reasonably addressed my comments, and I support publication

Reviewer #2

(Remarks to the Author)
The authors have addressed my concerns in a satisfactory manner. I therefore recommend publication of this manuscript.

Reviewer #3

(Remarks to the Author)
The authors have done a good job addressing the reviewers comments by adding additional experiments and clarifying aspects of the writing. Overall the manuscript is much improved and I am in favor of publication. Aspects of the manuscript could still be improved, particularly some aspects of the writing are still confusing.

For example, the abstract still says that "αTAT1 efficiently acetylates microtubules in expanded lattices and twisted tubulin states" whereas the results show and the discussion states that the expanded state is the predominant determinant of microtubule acetylation.

Another example is how the authors think that "the acetylation pattern of microtubules in cells" (line 19) is generated. ATAT1 shows low activity on GDP compacted microtubules (Figure 4). Thus ATAT1 is not expected to acetylate most microtubules in cells as they are in the compacted state (de Jager et al 2024).

The authors propose "One possible explanation is intermittent deacetylation by HDAC6." They should explain this idea more fully. In particular, this idea is based on the finding that overexpression of kinesin-1 results in excessive damage and a decrease in the amount of acetylation (Figure 3, Budaitis et al 2022, Andreu-Carbo et al 2024) presumably because ATA1 leaves the lumen and HDAC6 enters the lumen. Whether this possibility is pertinent in the absence of excessive damage is not clear to me.

The authors also propose "Alternatively, local MAP binding may induce lattice expansion and thereby increase effective αTAT1 activity," (lines 322-323). This possibility was already brought up in lines 316-318 so please rewrite the text to clarify. Importantly, this possibility has already been proposed in the field in the context of another tubulin modification, specifically lattice expansion is proposed to regulate where and how efficiently VASH1/SVBP acts to generate detyrosinated tubulin in cells (Yue et al 2023). The authors should properly acknowledge this previous work and put their work and ideas in context.

Open Access This Peer Review File is licensed under a Creative Commons Attribution 4.0 International License, which permits use, sharing, adaptation, distribution and reproduction in any medium or format, as long as you give appropriate credit to the original author(s) and the source, provide a link to the Creative Commons license, and indicate if changes were made.
In cases where reviewers are anonymous, credit should be given to 'Anonymous Referee' and the source.

The images or other third party material in this Peer Review File are included in the article's Creative Commons license, unless indicated otherwise in a credit line to the material. If material is not included in the article's Creative Commons license and your intended use is not permitted by statutory regulation or exceeds the permitted use, you will need to obtain permission directly from the copyright holder.
To view a copy of this license, visit https://creativecommons.org/licenses/by/4.0/

We thank the Editor and the Reviewers for their careful evaluation of our manuscript and for their constructive comments. We have revised the manuscript extensively in response to these suggestions and believe that the changes have strengthened the clarity and rigor of the study. Below, we provide a detailed, point-by-point response to all comments. All changes in the manuscript are highlighted.

Reviewer #1 (Remarks to the Author):

This paper addresses an important and currently intensely studied problem – the impact of microtubule lattice structure on post-translational tubulin modifications. The authors perform in vitro experiments with purified proteins to investigate the impact of microtubule damage and microtubule lattice conformations on acetylation of microtubules by alphaTAT and their deacetylation by HDAC6. The authors conclude that alphaTAT overrides the enzymatic activity of HDAC6, but is less active on strongly damaged microtubules. By comparing the acetylation efficiency of microtubules stabilized by different agents, the authors provide evidence that alphaTAT is sensitive to the expansion of the microtubule lattice.

If the data were of good quality, this study would have been an extremely useful addition to the field. Unfortunately, while the Western blots look good, the fluorescence microscopy and negative-stain TEM images are of poor quality, which makes the conclusions unreliable. The discussion of microtubule lattice conformation is superficial: it does not take into account that microtubules stabilized by different agents would have different protofilament numbers, which could strongly affect lattice twist. For this reason, the paper is not publishable in a peer-reviewed journal in its current form.

Specific comments:

1. Fluorescence images, for example in Fig. 1d, show many microtubule clumps; the patchy labeling for acetylated tubulin is not clearly visible. A comparison of the two channels — tubulin acetylation and total tubulin — along a single, well-resolved microtubule is needed for all conditions.

Following the reviewer's suggestion, we replaced the images in Fig. 1d with revised panels that first present an overview of the microtubule network together with the acetylated tubulin channel, followed by zoomed-in views of individual, well-resolved microtubules for all conditions. For the zoomed-in acetylation images, identical brightness and contrast settings were applied across conditions, enabling a direct visual comparison of acetylation levels; this information is now included in the figure legend. As microtubules are fixed prior to antibody staining, some degree of deformation or clumping is unavoidable, and this limitation is now explicitly stated in the Methods section.

2. Imaging and quantifications of microtubule damage by negative-stain TEM are very unconvincing. For example, it is intrinsically impossible to distinguish "broken MTs" from two adjacent seeds unless one observes them live under a fluorescence microscope before and after breaking. This type of damage should be excluded from quantification. Dimer-removal examples are not convincing, and it is completely unclear how any damage can be quantified in microtubule bundles, which predominate in some conditions. If the images are not convincing, the corresponding quantifications and ensuing conclusions are not meaningful. Importantly, cryo-ET is currently the preferred method to characterize lattice defects. For any analysis, quantifying microtubules that are not overlapping or bundled with other microtubules is essential.

We now include in Fig. 2 examples of individual microtubules for each damage class to clarify how damage types were identified. The Methods section ("Microtubule damage characterization") has been revised to describe the classification criteria in greater detail. Please note that we excluded microtubule bundles for our analysis; now stated in the Methods and figure legends.

We agree that EM cannot distinguish adjacent seeds from true breaks. Accordingly, microtubules were classified as "broken" only when two ends were <100 nm apart, consistent with a kink-induced break. We analysed all conditions using the same approach which highlights the difference between conditions. We apologize that this criterion and its limitations were not clearly stated previously; they are now added to the Limitations of the Study and the Method section. We also renamed "broken MT" to "break".

Negative-stain EM has been used to visualize and quantify missing dimers along the microtubule lattice (e.g. Reid et al 2017, Budaitis et al. 2022, Vemu et al. 2018). In comparison to these published studies, our negative-stain data are of sufficient quality to identify dimer dissociation (see comparison between literature and our data below (Fig. 1 below, Fig. 2 in manuscript). Dimer dissociation observed in negative stain are also visible by cryo-ET which we now performed for the revision, although the throughput is inherently lower and imaging time more costly, which would not justify the analysis. Representative cryo-ET images have been added to the supplementary material (Fig. S2b) for comparison.

a

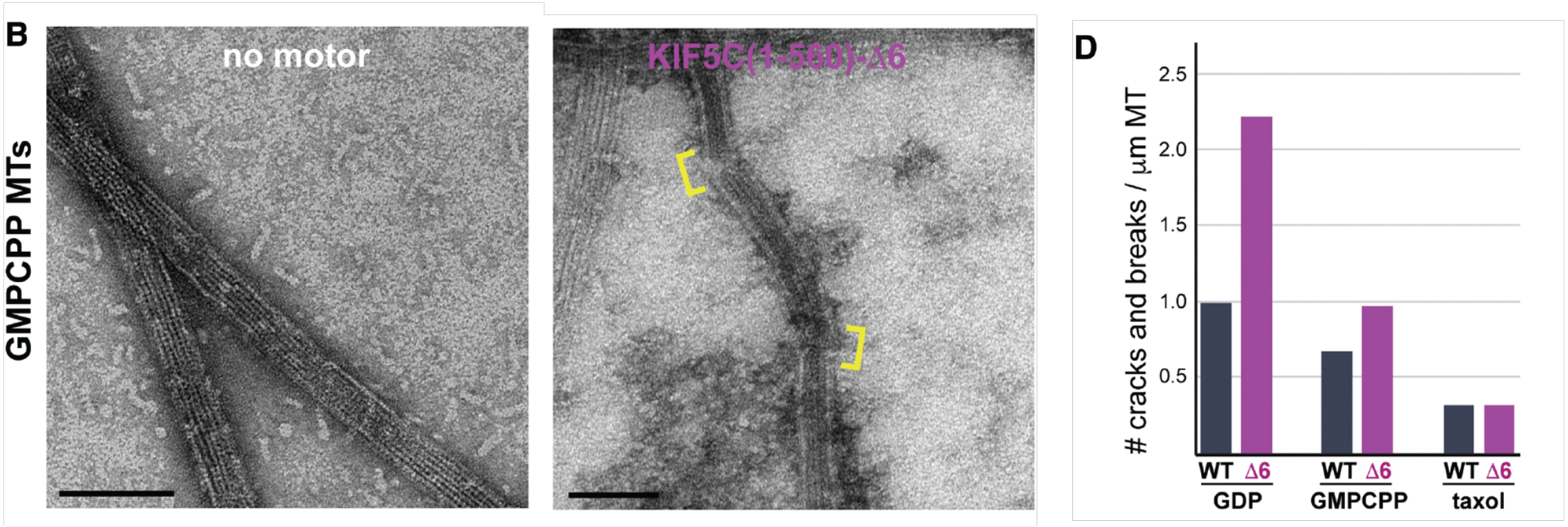

b

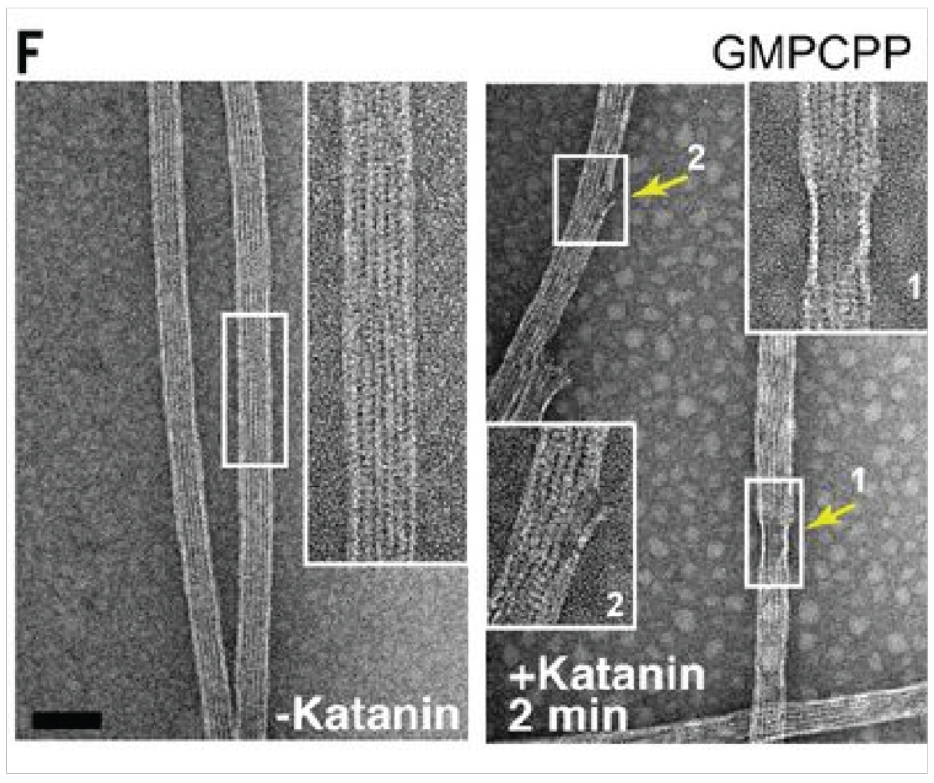

c

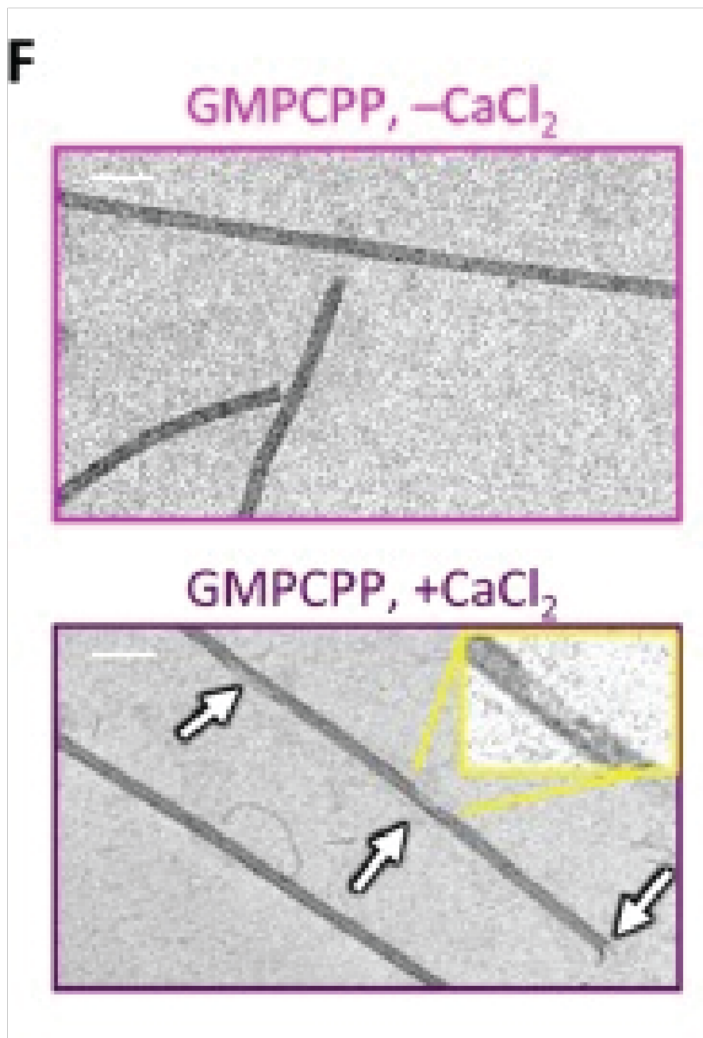

Fig. 1. Comparison of published negative stain electron microscope images of microtubule damage. **a** KIF5C control or Δ6mutant-induced damages on GMPCPP microtubules with quantifications of cracks and breaks. Taken from Budaitis et al. 2022. **b** Katanin-induced damages on GMPCPP microtubules. Taken from Vemu et al. 2018. **c** Control or $CaCl_2$-induced damages on GMPCPP microtubules. Taken from Reid et al. 2017.

3. The discussion about lattice twist seems superficial and incorrect — GMPCPP-stabilized microtubule seeds in vitro typically have 14 protofilaments (and thus a negative twist). The authors should quantify the protofilament numbers of the different stabilized microtubule preparations they study and discuss their conformations taking these data into consideration.

Following this suggestion, we quantified the protofilament number of the different stabilized microtubule preparations (GMPCPP, taxol, and peloruside A; new panel in Fig. 4b). As expected, GMPCPP-stabilized microtubules predominantly adopt a 14-protofilament conformation with a negative lattice twist; this has now been corrected in the manuscript. Under peloruside A conditions approximately 80% of microtubules also exhibit a 14-protofilament architecture.

We also added a literature-based table summarizing reported protofilament numbers, dimer rise, and lattice twist values for the different stabilization conditions (Fig. 4b). These additions do not change our overall conclusion that dimer rise, rather than lattice twist alone, is the primary determinant of αTAT1-mediated acetylation efficiency.

Reviewer #2 (Remarks to the Author):

In this manuscript the dynamics of microtubule (MT) acetylation of lysine 40 of α-tubulin, a hallmark of stable MTs in cells, is investigated in vitro. The authors generate MTs with different properties (conformations and/or damage) and either add α-tubulin acetyltransferase 1 (ATAT1), the enzyme that generates this mark, or histone deacetylase 6 (HDAC6), which removes acetylation, or both enzymes. By modifying MT lattice conformation and introducing damage in defined manners the authors show that the acetylation efficiency of ATAT1, which overwrites HDAC6 enzymatic activity, decreases when MTs are damaged. They also find that ATAT1 efficiently acetylates MTs in expanded lattices and twisted tubulin states, whereas compacted lattices reduce ATAT1 activity.

Given the cellular importance of lysine 40 acetylation of α-tubulin (in short: MT acetylation) the activities and functions of ATAT1 and HDAC6 have been examined by different groups. For example, the fact that ATAT1 prefers expanded MTs has been published (Ref 63). The main novelty of the present manuscript appears to be the finding that ATAT1 is sensitive to MT lattice integrity. I believe the results reported here are of importance for the field. I do have several comments, which I list below.

Comments.

1) In Fig. 1 ATAT1 and HDAC are added at defined concentrations, either alone or in combination. It would be interesting to use different enzyme concentrations. For example, is the roughly 5-fold increase observed with ATAT1 in Fig. 1b, c, a saturation effect or can this be further increased using higher concentrations of ATAT1? In Fig. 1c in the competition experiment a ratio of 0.1 ATAT1 to 1 HDAC6 is used. This leads to a 1.35-fold increase in acetylated tubulin. This experiment should be repeated with 0.1 ATAT1 alone, to see if there was any competition by HDCA6.

Following the reviewer's suggestion, we tested a range of αTAT1 concentrations (0, 0.1, 0.5, 1, 2, and 5), while keeping HDAC6 and tubulin concentrations constant (new Fig. S1a). These data show that the ~5-fold increase in microtubule acetylation observed in Fig. 1b,c reflects saturating conditions, as further increases in αTAT1 concentration do not result in a substantial additional increase in acetylation.

We also performed the suggested control experiment using 0.1 αTAT1 in the absence of HDAC6. This experiment yielded unexpected results. Under these conditions, αTAT1 alone was insufficient to induce detectable microtubule acetylation within 60 min, whereas the same αTAT1 concentration in the presence of HDAC6 resulted in a 1.35-fold increase in acetylated tubulin. These results indicate that HDAC6 enhances αTAT1-mediated acetylation at low αTAT1 concentrations. At higher αTAT1 concentrations (>0.5), microtubule acetylation appears independent of HDAC6 (Fig. 1c, Fig. S1a).

2) In Figures 2 and 3 visualization of MT damage, induced by snap freezing or mutant kinesin, by EM is shown. Four classes of damage are described by the authors, i.e dimer dissociation, kink, sheet

opening and broken MTs. However, the “dimer removal” is very difficult to see in Fig. 2a and seems impossible to quantify accurately, also given the density of MTs in EM images (shown in Fig. 2d). Hence, the 14% dimer dissociation along the lattice (in Control) is likely an underestimation, and the other two values therefore an overestimation. Nevertheless, the qualitative effect of the damaging treatments seems clear. In Fig. 2e dimer removal is supposed to be indicated in blue and sheet opening in orange, however, the percentages associated with blue (43%) and orange (14%) in Control suggest the opposite, i.e. dimer removal is orange and sheet opening blue. If I am correct, the authors should correct this and carefully check their panels/labelling.

In response to this comment, we added representative zoomed-in examples of each microtubule damage class used for the analysis. For damage classification, EM images were analyzed in regions of the grids with lower microtubule density than in the original Fig. 2d, and the analysis was restricted to non-bundled microtubules. The Methods section now provides a more detailed description of the criteria used to identify and classify the different damage types.

In addition, the labeling inconsistency in Fig. 2e has been corrected. To improve clarity, the damage-type distributions for the different conditions are now presented as stacked bar graphs instead of pie charts in the revised Fig. 2e.

3) In Fig. 2b, c there is a condition called "No". I assume this means that no mutant kinesin was added, and that it is in essence a control. If so, I understand the same number of events per micron for "No" (0.15) and "Control" (0.14) in Fig. 2b. However, how can the values differ in Fig. 2c?

To improve clarity, we renamed the condition previously labeled “No” to “Control Kinesin-1Δ6” in Fig. 2. This control condition about twofold more sheet-opening events than the snap-freezing control (0.04 vs. 0.02 events/µm). This difference likely reflects the distinct incubation conditions used for the two controls. The kinesin control was subjected to the same incubation protocol as the mutant kinesin samples: GMPCPP-stabilized microtubules (3.5 µM tubulin) were supplemented with 2.7 mM ATP in 1× BRB and incubated for 15 min at 37 °C prior to blotting. By contrast, the snap-freezing control was directly blotted. These distinctions are now explicitly described in the figure legend and the Methods section.

4) I do not understand the y-axis in Fig. 2c "Percentage of microtubule in sheet opening state observed along microtubules by EM for both damaging conditions". Could the authors explain this in more detail?

To address this point, we clarified the description of the y-axis in now Fig. 2d by revising both the axis label and the figure legend. The y-axis is now presented as a fraction: “Fraction of total microtubule length in an open-sheet configuration (sum of sheet-opening lengths divided by total microtubule length), based on EM quantification for both damaging conditions.” In addition, this explanation has been added to the Methods section.

5) The experiments in Fig. 3 were done with either ATAT1 or HDAC6. Results in Fig. 3c suggest that large sheet openings caused by snap freezing enhance HDAC6 access to the luminal K40 site. Given the

higher activity of HDAC6 it would be interesting to do a competition experiment as shown in Figure 1, also with higher concentrations of ATAT1.

To test this possibility, we performed additional competition experiments on snap-frozen microtubules and now present these data in Fig. 3c,d. Figure 3c shows a direct competition between αTAT1 and HDAC6, analogous to the experiment shown in Fig. 1. "In contrast to intact microtubules, where equimolar αTAT1 and HDAC6 resulted in a ~5-fold increase in acetylation (Fig. 1c), snap-frozen microtubules showed a net ~6-fold reduction in acetylation relative to untreated microtubules. This corresponds to an approximately 30-fold difference in acetylation levels between intact and damaged microtubules and indicates that HDAC6 activity dominates in damage conditions. Reducing the HDAC6 concentration 10-fold while keeping αTAT1 constant partially alleviated this effect, resulting in a reduced but ~2-fold increase in acetylation.

Consistent with these findings, increasing αTAT1 concentration up to 50-fold at constant HDAC6 level did not measurably increase acetylation on snap-frozen microtubules (Fig. 3d). Together, these data show that αTAT1-mediated acetylation is effectively low on snap-frozen microtubules in the presence of HDAC6. Although αTAT1 retains some capacity to acetylate snap-frozen microtubules — albeit at reduced efficiency compared to intact lattices (Fig. 1b-c, 3c-d) — its activity is outweighed by HDAC6-mediated deacetylation (Fig. 3c-d). Given the markedly higher catalytic efficiency (40000-fold k_{cat}/K_M) of HDAC6 toward free tubulin[54] compared to αTAT1 acting on polymerized microtubules[62], increased access to the microtubule lumen generated by snap freezing likely shifts HDAC6 activity toward a regime closer to that observed for free tubulin. Consequently, even a 50-fold excess of αTAT1 is insufficient to counterbalance HDAC6 activity in damaged microtubules."

6) Fig. 3c tries to visually explain the results obtained in Figure 3. However, while the more efficient entry of HDAC6 is represented in the panel, the reduction in enzymatic activity of ATAT1 due to damage, is not well seen. I would suggest to either remove this panel or to try and improve it.

We moved the panel to the supplementary, updated the panel including a decrease in aTAT1 enzymatic activity at damage sites, now indicated by yellow hollow pentagon symbols for the absence of Ac and those sites are indicated with red arrowheads (Fig. S2c).

7) On page 7, lines 201-204, the authors state "Notably, GMPCPP-microtubules - having the most expanded lattice - were slightly more acetylated than taxol-stabilized ones. The intermediate GMPCPP/GDP state showed less increase in acetylation with 3.05-fold, and a GDP-compacted microtubule lattice led to the lowest acetylation increase of only 1.80-fold". I am not a statistician but can such conclusions be drawn in the absence of statistical significance for some of the results (GMP vs Taxol: 0.9640, GMP vs GMP/GDP: 0.2673)?

Indeed, conclusions should be limited to statistically supported comparisons. We therefore removed the statement comparing GMPCPP- and taxol-stabilized microtubules and retained only the statistically significant comparison between GMPCPP and GMPCPP/GDP microtubules.

8) Fig. 4b tries to visually explain MT conformations in the presence of different compounds, and their effects on acetylation. What is the value of panel 4b? Why present it before Fig. 4c?

We now rearranged the Fig.4 to include a new analysis of protofilament number and replaced the value panel with a literature-based table summarizing reported protofilament numbers, dimer rise, and lattice twist values for the different stabilization conditions.

9) In Fig. 4c the authors show a "representative" western blot (one of four experiments performed). However, in the "protein load" samples, the last two samples (Taxol, Peloruside A) have considerable less protein loaded. Since this is a representative result, how is this possible? How does this affect the quantification shown below the blot?

Western blot images were exchanged, now displaying similar protein load. Note that western blot quantifications were performed by dividing the intensity of the acetylation signal by the amount of protein loaded, as described in the Methods. Thus, differences in absolute protein load below pixel saturation do not affect quantification comparison here.

10) In the Methods section the authors mention that GMPCPP-stabilized seeds contain both Taxol (1 micromolar) and GMPCPP (0.5 mM). Since Taxol and GMPCPP influence acetylation I wonder how these compounds are removed in subsequent experiments where seeds are used.

Microtubule seeds were used in all conditions for Fig. 4a, to ensure that the effects of the compounds on acetylation levels were comparable across experiments. In the final reaction mixture, the addition of seeds resulted in final concentrations of 20 nM taxol and 10 µM GMPCPP. This information has now been added to the Methods section: "...incubated with 0.2 µM microtubule seeds (final concentrations in the reaction mix: 20 nM taxol, 10 µM GMPCPP) in 1x BRB80". We furthermore clarify in the main text that GMPCPP- and taxol-stabilized seeds were used in all conditions.

11) On page 9, lines 278-279, the authors state: "Acetylation could specifically mark stable microtubules that adopt an expanded lattice state". I thought that the long-lived nature of stable MTs explained increased acetylation. How did the authors reach the conclusion that stable MTs adopt an expanded lattice state?

Long-lived microtubules are correlated with increased acetylation, but acetylation is not uniform along the lattice and instead occurs in discrete segments. This suggests that microtubule lifetime alone is insufficient to explain acetylation patterns. One possibility is that regions in-between segments are deacetylated by HDAC6. An alternative possibility is that local changes in lattice conformation—potentially induced by MAP binding—enhance αTAT1 effective activity, similar to the expanded lattice state of GMPCPP-stabilized microtubules, which show higher acetylation levels (Fig. 4a). We have revised the Discussion to clarify this interpretation

Reviewer #3 (Remarks to the Author):

The manuscript by Egoldt and colleagues investigates the mechanism by which acetylated tubulin is generated in microtubules by the enzymes aTAT and HDAC6. The authors show that enzymatic activity is sensitive to damage sites in the microtubule lattice, the expanded vs compacted state of the microtubule lattice, and the twist of the microtubule lattice. The work is important for the field but

seems a bit preliminary as there are caveats and alternative explanations that could be addressed experimentally but should at a minimum be addressed in the text. The manuscript requires further text edits and image presentation to improve clarity and readability.

Major issues:

1) Figure 1 legend title “αTAT1 overwrites HDAC6 activity on intact microtubules in vitro“ and lines 106-107 “HDAC6 deacetylation activity is low in microtubules so that the presence of its counteractor αTAT1overwrites its activity.” This seems to be a mis-interpretation of the data. HDAC activity is low on microtubules (Figure 1b) so how can its counteractor aTAT “overwrite” it? Figure 1c basically shows that adding an equimolar amount of HDA6C (1:1 A:H ratio) or a bit of HDAC6 (1:0.1 A:H ratio) has no effect on AcTub levels. The only parameter that changes the amount of AcTub is to lower the amount of aTAT (0.1:1 A:H ratio). Thus, the proper interpretation would be that the presence of HDAC6 does not have any effect (positive or negative) on aTAT activity.

We agree that the original statement was not well worded, indeed HDAC6-mediated deacetylation of microtubules is low. We have revised the figure legend and corresponding text accordingly. In addition, following the suggestion of Reviewer 2, we performed additional experiments that add new factors to the original picture. These data show that at low αTAT1 concentrations, the presence of HDAC6 enhances αTAT1-mediated acetylation efficiency (new Fig. 1d).

Figure 1d,e,f: since the AcTub antibody staining in this microscopy assay is inconsistent across samples (depending on what proteins are added to the assay), these data should be restricted to the supplement

Figure 1d seems to be low quality and thus difficult to visualize. In general, it is helpful to have microscopy images large and graphs small. Also, what are the boxes in the AcTub column for?

To address these points, we improved the quality of the microscopy images in now Fig. 1e and added zoomed-in views of individual, well-resolved microtubules for all conditions. For the zoomed-in acetylation images, identical brightness and contrast settings were applied across conditions, enabling a direct visual comparison of acetylation levels; this information is now included in the figure legend.

2) Figure 2 is difficult to parse with so many graphs and colors. The pie charts in 2e are particularly difficult to compare (a stacked bar graph may be better?) and basically show the same data as in 2f. Please think about how to better display this data. Perhaps separating the kinesin-1d6 and snap freezing data into different sections/graphs?

Thank you for your suggestions. We have removed the pie charts in Fig. 2 and replaced them with a stacked bar graph. In addition, we revised the color scheme and reorganized the panel layout to improve clarity and readability of the figure overall.

The criteria for each of these types of damage need to be clearly defined in the methods section. Particularly for dimer removal, this is not at all apparent from the image in 2a.

The criteria used to define each type of microtubule damage are now described in more detail in the Methods section (“Microtubule damage characterization”). In addition, we added representative zoomed-in examples of each damage class used for the analysis.

The types of damage under each condition in 2d should be marked with the same color arrows as shown in 2a.

Fig. 2d has been moved to Supplementary Fig. S2. The color coding of arrows used to indicate the different damage classes is now applied consistently throughout the manuscript.

How does the damage observed in these experiments compare to previous work using negative stain EM (Budaitis et al 2023, Reid et al 2017, Gonzalez et al 2024)?

The damage observed in our experiments is qualitatively consistent with previous reports using negative-stain EM, as illustrated by the comparison of figures shown below (Fig.1). While the absolute damage frequencies measured in our experiments are generally lower in both control (0.15–0.5 events/µm) and treatment conditions (0.5–1 events/µm) compared to those reported by Budaitis et al., the level of damage observed in our control samples is comparable to values reported in other studies (0.27±0.09 defects/µm Triclin et al 2019; 0.07 breakage/µm Reuther et al 2025 (preprint). We now cite the references accordingly.

a

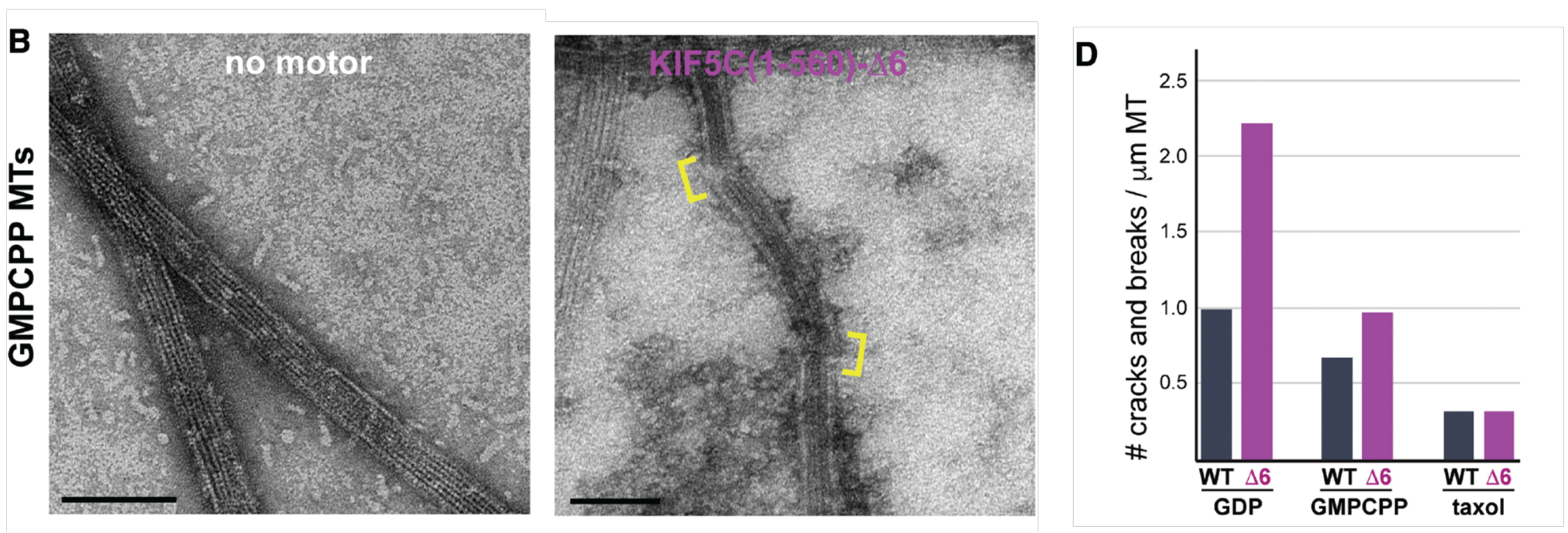

b

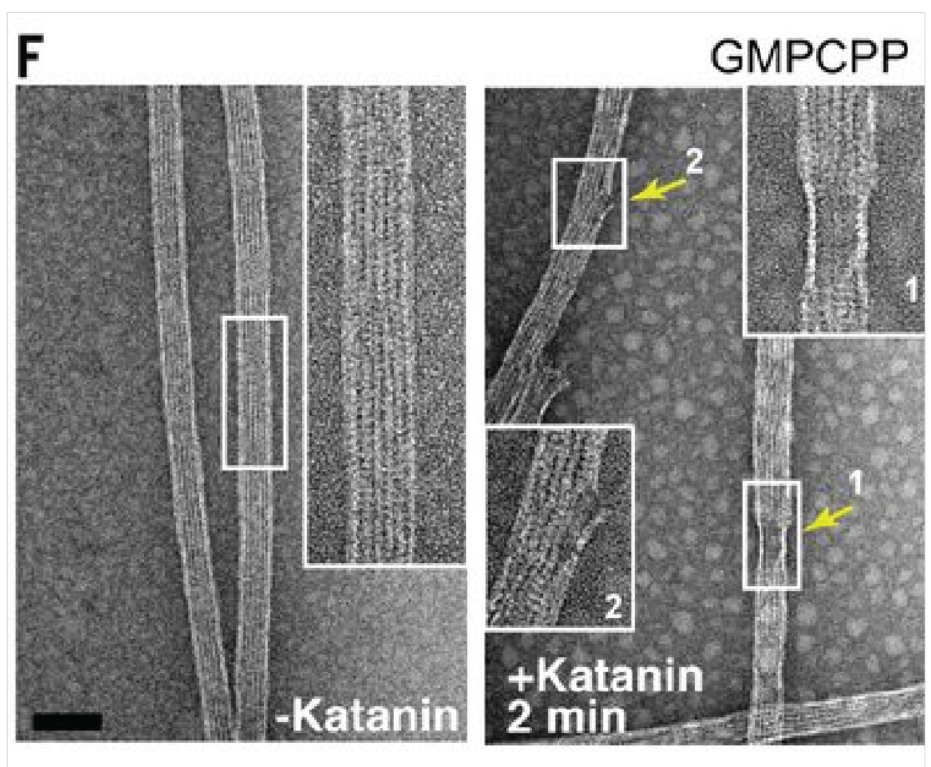

c

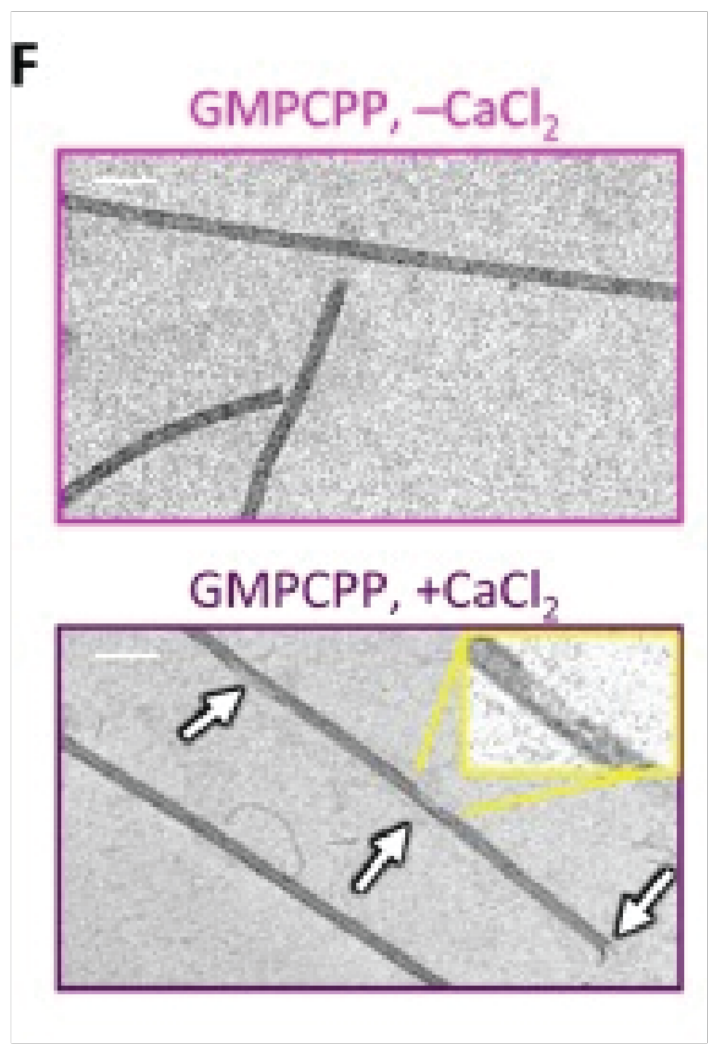

3) For figure 3, an alternative possibility is that the damage sites permit aTAT to leave the microtubule lumen before it can generate AcTub and this is why there is less AcTub in the snap freeze and kinesin-1d6 damaged microtubules. At a minimum, this need to be discussed in the text.

We agree with this interpretation and did add this aspect to the main text.

4) The authors suggest that tubulin compaction vs expansion in the lattice regulates An alternative way to show that aTAT is sensitive to lattice expansion is to use the same microtubules (e.g .GTP or GMPCPP/GTP mix) and add a MAP that is known to expand the lattice such as MAP7 or the kinesin-1 rigor versus a MAP that is known to compact the lattice such as Tau or MAP2. Indeed, these methods were already used in the field to examine how expansion vs compaction influences tubulin detyrosination and the authors should mention this work (Yue et al 2023).

In the present study, our primary focus was on how lattice damage alters αTAT1 and HDAC6 activity. To interpret these findings, we extended the analysis to lattice conformation effects on acetylation and therefore chose well-controlled and extensively characterized systems—drug-stabilized microtubules and GMPCPP—where detailed ultrastructural data are available. Extending this analysis to MAP-mediated lattice modulation would be valuable but represents a substantial additional direction beyond the scope of the current work. We therefore did not pursue these experiments here but now mention and cite the relevant study by Yue et al. (2023).

The authors also suggest that differences in microtubule twist are responsible for the differences in aTAT activity. I find this an interesting possibility but since this is the first report (to my knowledge) of how microtubule twist affects a microtubule modifying protein, the authors needs to be more thorough. What we know about microtubule twist is based on selected EM images and the twist of microtubules not included in these measurements is unknown. That is, we don't really know the full extent of twist in the lattice under these different experimental conditions. The possibility that the effects are due to differences in protofilament number is mentioned in the Discussion but most microtubules are a mixture of protofilament numbers so unless the authors know the distribution of protofilament # microtubules across their conditions, this cannot be discounted.

We agree that the role of microtubule lattice twist in regulating αTAT1 activity requires careful interpretation, given current limitations in quantifying twist across entire microtubule populations. To address this, we quantified the protofilament number of the stabilized microtubules used in this study (GMPCPP, taxol, and peloruside A; new panel in Fig. 4b), as protofilament number provides an experimentally accessible proxy for lattice twist. As expected, GMPCPP-stabilized microtubules predominantly adopt a 14-protofilament conformation with a negative twist, and under peloruside A conditions approximately 80% of microtubules also exhibit a 14-protofilament architecture.

We also added a literature-based table summarizing reported protofilament numbers, dimer rise, and lattice twist values for the different stabilization conditions (new panel in Fig. 4b). While these additions do not capture the full distribution of lattice twist states, they explicitly account for protofilament heterogeneity and support our conclusion that lattice spacing (dimer rise), rather than lattice twist/PF number, is the primary determinant of αTAT1-mediated acetylation efficiency.

Minor issues:

I am curious why the authors chose snap-freezing to generate damaged microtubules as opposed to exposing GMPCPP microtubules to $CaCl_2$ (Gonzalez et al 2024) or generating open lattices using GTPgS (Reid et al 2017).

We tested several approaches to reproducibly induce microtubule damage while maintaining the polymerized state, including treatment with $CaCl_2$ and H_2O_2. $CaCl_2$ caused substantial depolymerization of GMPCPP-stabilized microtubules in addition to local damage, as observed by TIRF microscopy, and was therefore excluded. Other approaches, including H_2O_2 treatment, did not yield reproducible local damage under our experimental conditions and were not pursued further. GTPγS was also not considered, as it does not nucleate microtubules without GMPCPP seeds and does not provide a strategy to locally damage GMPCPP-stabilized microtubules, as achieved by snap freezing.

The manuscript text should state that the seeds are GMPCPP- and taxol-stabilized.

We included this information in the manuscript text.

Lines 135-136: "as wildtype kinesin-1 damages stabilized microtubules insufficiently (see more details in Methods)." I could not find any additional details about this in the methods section.

We removed the reference to additional methodological details and instead added the appropriate citation to Budaitis et al. (2023).

Figure 4b needs a heading for each column.

We modified Figure 4b, see the comment above.

REVIEWERS' COMMENTS:

Reviewer #1 (Remarks to the Author):

The authors have reasonably addressed my comments, and I support publication

Reviewer #2 (Remarks to the Author):

The authors have addressed my concerns in a satisfactory manner. I therefore recommend publication of this manuscript.

Reviewer #3 (Remarks to the Author):

The authors have done a good job addressing the reviewers comments by adding additional experiments and clarifying aspects of the writing. Overall the manuscript is much improved and I am in favor of publication. Aspects of the manuscript could still be improved, particularly some aspects of the writing are still confusing.

Thank you for pointing out confusions in writing; we addressed your concerns accordingly.

For example, the abstract still says that “αTAT1 efficiently acetylates microtubules in expanded lattices and twisted tubulin states” whereas the results show and the discussion states that the expanded state is the predominant determinant of microtubule acetylation.

We removed the statement of twisted tubulin states in the abstract.

Another example is how the authors think that “the acetylation pattern of microtubules in cells” (line 19) is generated. ATAT1 shows low activity on GDP compacted microtubules (Figure 4). Thus ATAT1 is not expected to acetylate most microtubules in cells as they are in the compacted state (de Jager et al 2024).

We changed the wording to focus on dynamic transitions between compacted and expanded lattices in cells.

The authors propose “One possible explanation is intermittent deacetylation by HDAC6.” They should explain this idea more fully. In particular, this idea is based on the finding that overexpression of kinesin-1 results in excessive damage and a decrease in the amount of acetylation (Figure 3, Budaitis et al 2022, Andreu-Carbo et al 2024) presumably because ATA1 leaves the lumen and HDAC6 enters the lumen. Whether this possibility is pertinent in the absence of excessive damage is not clear to me.

The authors also propose “Alternatively, local MAP binding may induce lattice expansion and thereby increase effective αTAT1 activity,” (lines 322-323). This possibility was already brought up in lines 316-318 so please rewrite the text to clarify. Importantly, this possibility has already been proposed in the field in the context of another tubulin modification, specifically lattice expansion is proposed to regulate where and how efficiently VASH1/SVBP acts to generate detyrosinated tubulin in cells (Yue et al 2023). The authors should properly acknowledge this previous work and put their work and ideas in context.

We revised the Discussion to explain the idea of intermittent HDAC6-mediated deacetylation more clearly. We also note that, because our experiments were performed with GMPCPP-stabilized microtubules, motor activity on GDP-microtubules may generate larger lattice defects and therefore greater enzyme exchange, as micrometer long incorporation sites are reported in the literature for in vitro and cell studies.

In addition, we rewrote the section on MAP-induced lattice expansion to remove redundancy and to better integrate this idea with the damage-dependent effects on αTAT1 activity. We now also cite Yue et al. (2023) and place our interpretation in the broader context of structural regulation of tubulin-modifying enzymes.
