# Supplementary material for: Microtubule lattice conformation and integrity regulate α-tubulin acetylation
Source: Commun Biol. 2026 Jun 8;9:1211. doi: 10.1038/s42003-026-10157-4 (PMC13582946; doi:10.1038/s42003-026-10157-4)
Supplement: Supplementary file 2 [file 42003_2026_10157_MOESM2_ESM.pdf]

SUPPLEMENTARY INFORMATION

for

Microtubule lattice conformation and integrity regulate α-tubulin acetylation

Cornelia Egoldt[1], Marie-Claire Velluz[1], Joshua Tran[1] and Charlotte Aumeier[1]

[1]Department of Biochemistry, University of Geneva, 1211 Geneva, Switzerland

Corresponding author: charlotte.aumeier@unige.ch

SUPPLEMENTARY FIGURES

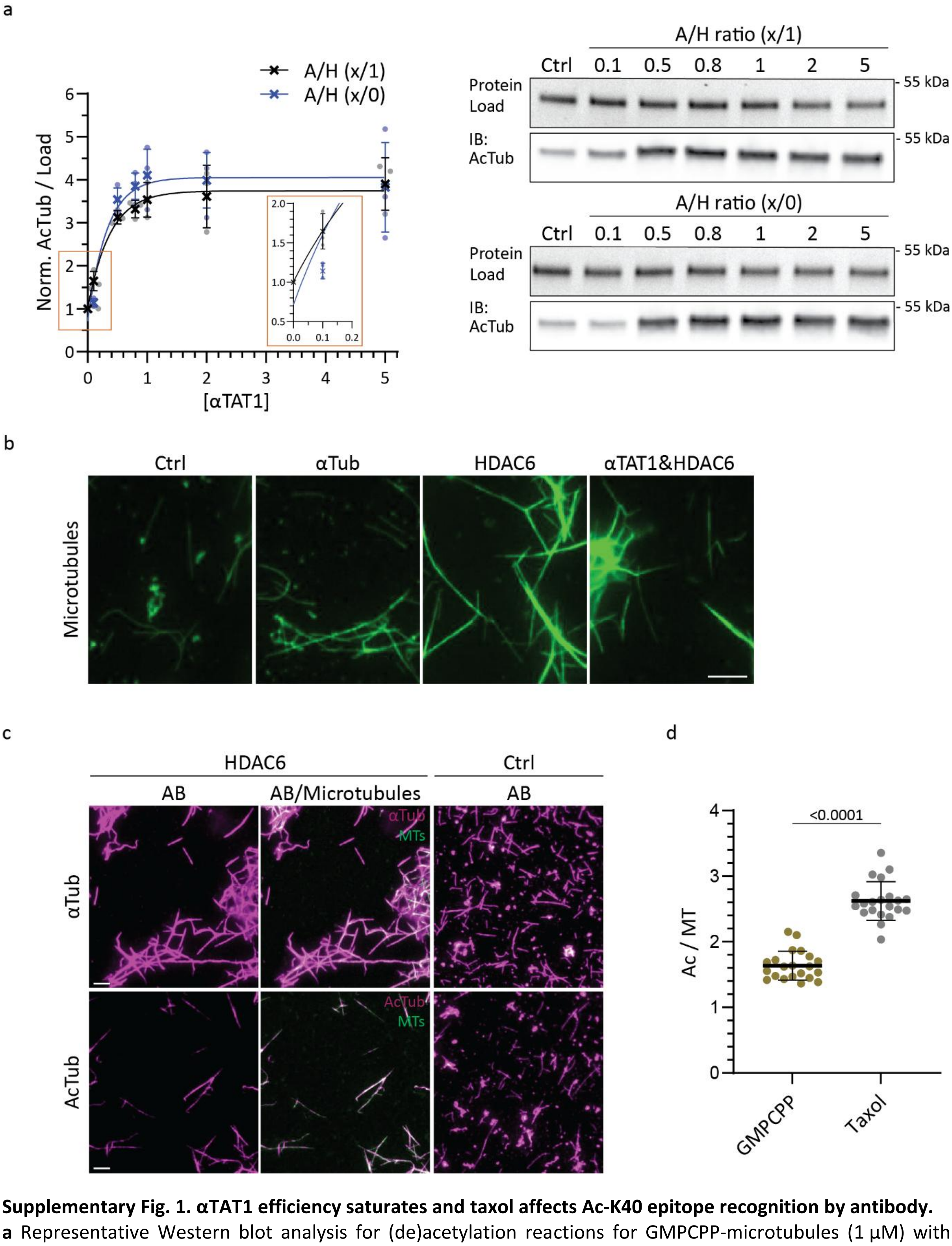

Supplementary Fig. 1. αTAT1 efficiency saturates and taxol affects Ac-K40 epitope recognition by antibody.
a Representative Western blot analysis for (de)acetylation reactions for GMPCPP-microtubules (1 µM) with different A/H (αTAT1/HDAC6) ratios with increasing αTAT1 concentrations (0.1-5 µM) in the absence (blue) or presence of 1 µM HDAC6 (black). The orange boxed area shows a zoom-in of the initial linear signal increase. Quantifications of AcTub levels relative to the protein load, from three (black) or four (blue) independent experiments. Statistics: one-way ANOVA. Mean with SD. **b, c** Representative immunofluorescence images of fluorescent microtubules (green) that were incubated with (**b**) αTAT1, HDAC6, both enzymes or control conditions and fixed; or (**c**) with HDAC6 or control conditions, fixed and immunostained with alpha-tubulin (αTub) or acetylated tubulin (AcTub) antibodies (AB) (magenta). Scale bars: 5 µm. **d** Immunofluorescence signals

of acetylated tubulin over microtubules for GMPCPP (n=21) or taxol (n=21) in control conditions from one experiment. Statistics: two tailed t test. Mean with SD.

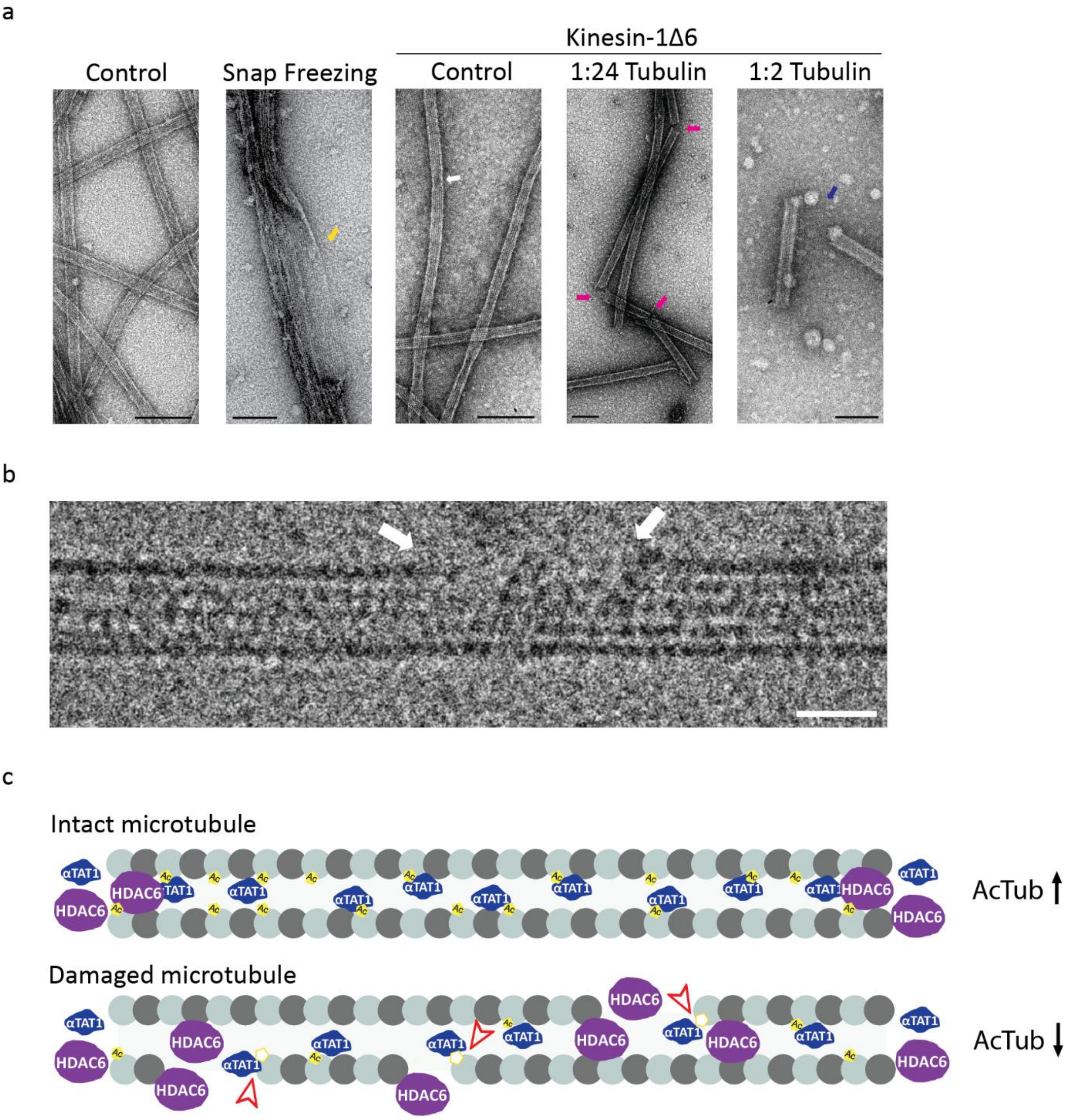

Supplementary Fig. 2. Distinct damage phenotypes for microtubule damaging conditions. a Representative EM images for different damaging conditions. Yellow arrows - "sheet openings", magenta arrows - "kink" events, white arrow - "dimer dissociation" and blue arrow - "break". Scale bars: 100 nm. **b** Cryo electron tomography image of dimer dissociation from the microtubule lattice indicated with white arrows. Scale bar: 20 nm. **c** Cartoon depicting acetylation levels in the presence of αTAT1 (blue) and HDAC6 (magenta) of intact microtubules with increased acetylation (above) and damaged microtubules showing decreased acetylation (below). Without damage, αTAT1 diffuses through the microtubule and acetylates stochastically, while HDAC6 entry is limited. Acetylation sites are labeled with yellow pentagon symbols in the microtubule lumen. The absence of acetylation is marked by hollow yellow pentagon symbols, and sites are indicated with red arrow heads. Damage along the shaft facilitates HDAC6 and αTAT1 access to the lumen, but αTAT1 acetylates only intact microtubules efficiently.

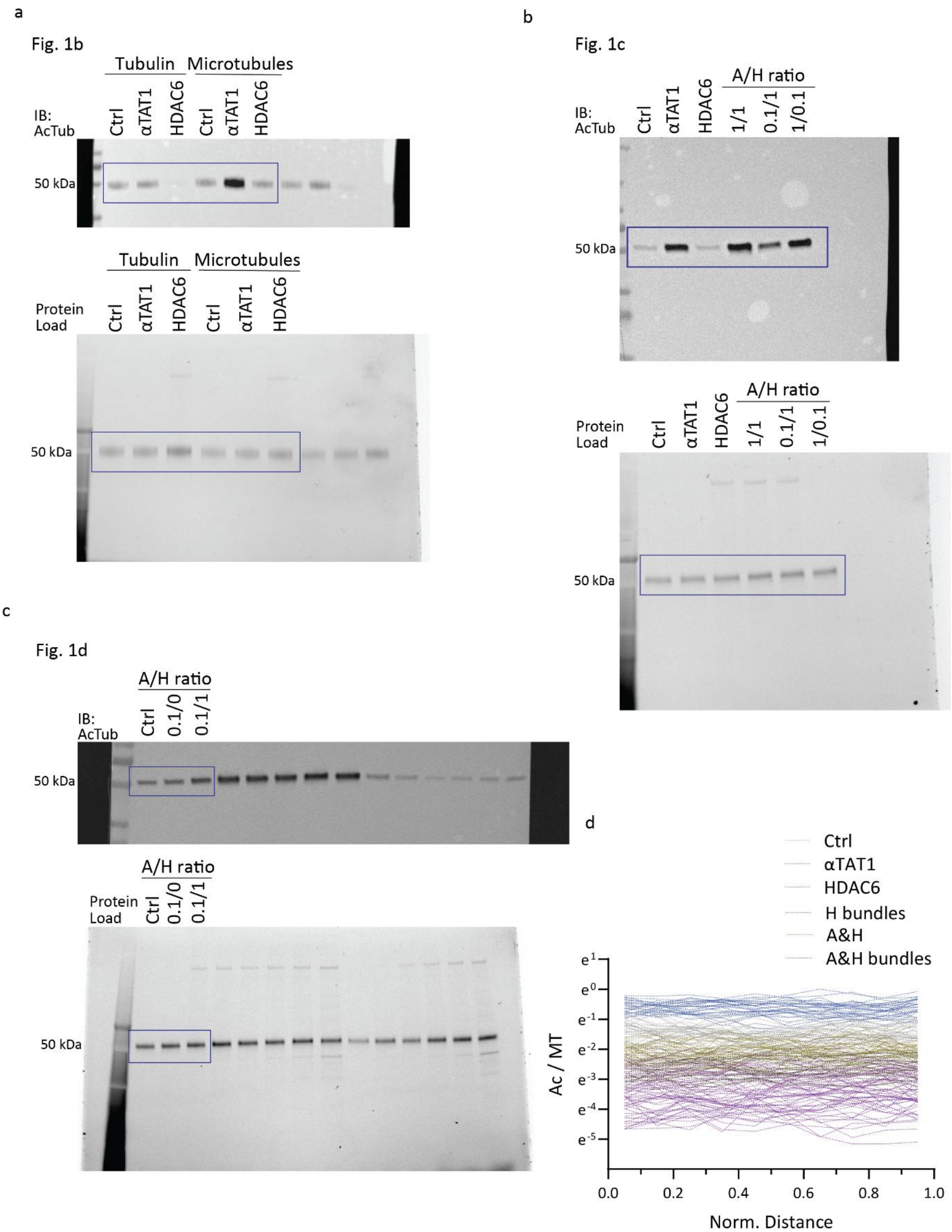

Supplementary Fig. 3. Raw data related to Fig. 1. a-c Uncropped Western Blot images and UV-exposed gels for protein load for (**a**) Fig. 1b, (**b**) Fig. 1c, (**c**) Fig. 1d. **d** Individual data for Fig. 1g.

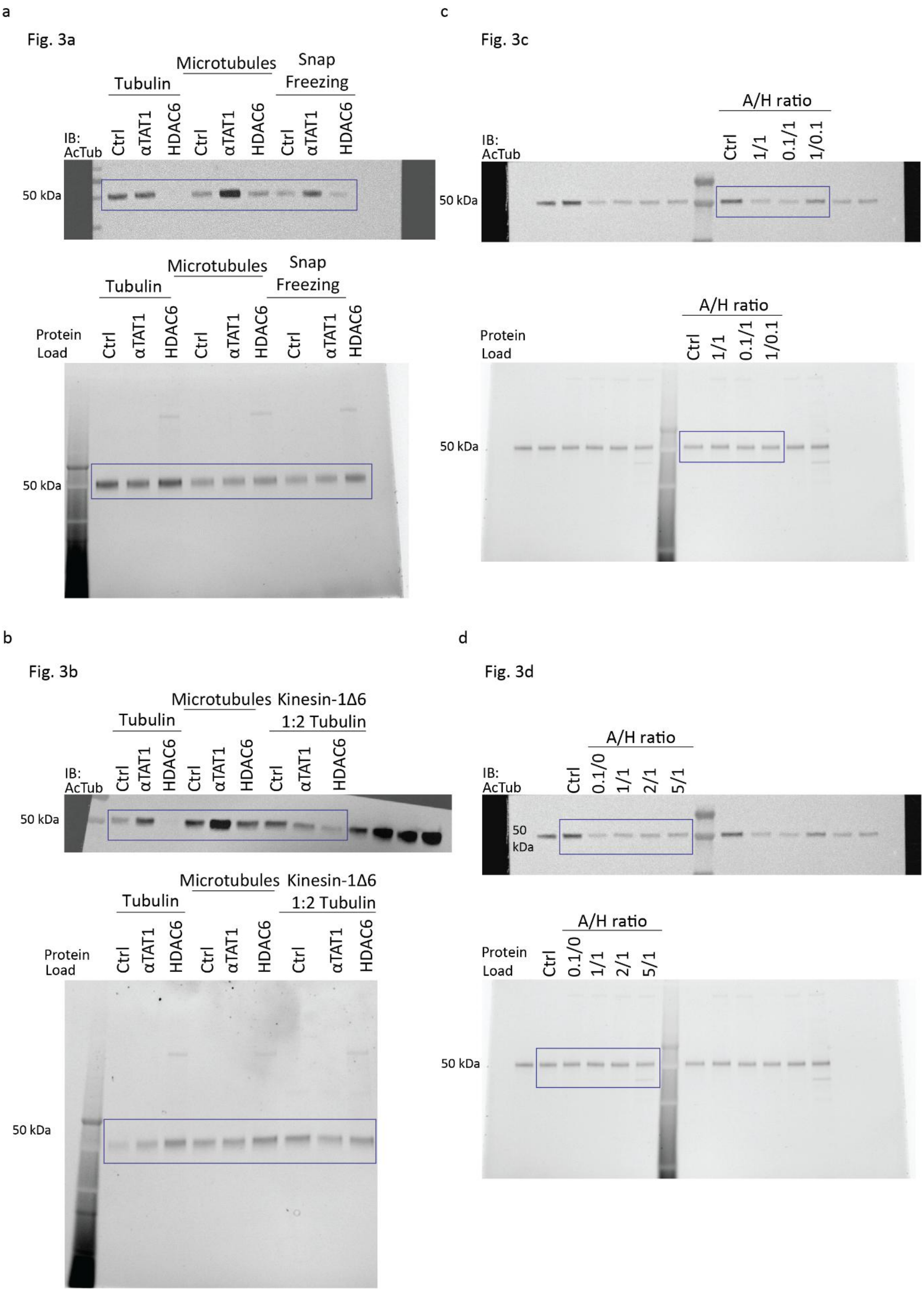

Supplementary Fig. 4. Raw data related to Fig. 3. a-d Uncropped Western Blot images and UV-exposed gels for protein load for (**a**) Fig. 3a, (**b**) Fig. 3b, (**c**) Fig. 3c and (**d**) Fig. 3d.

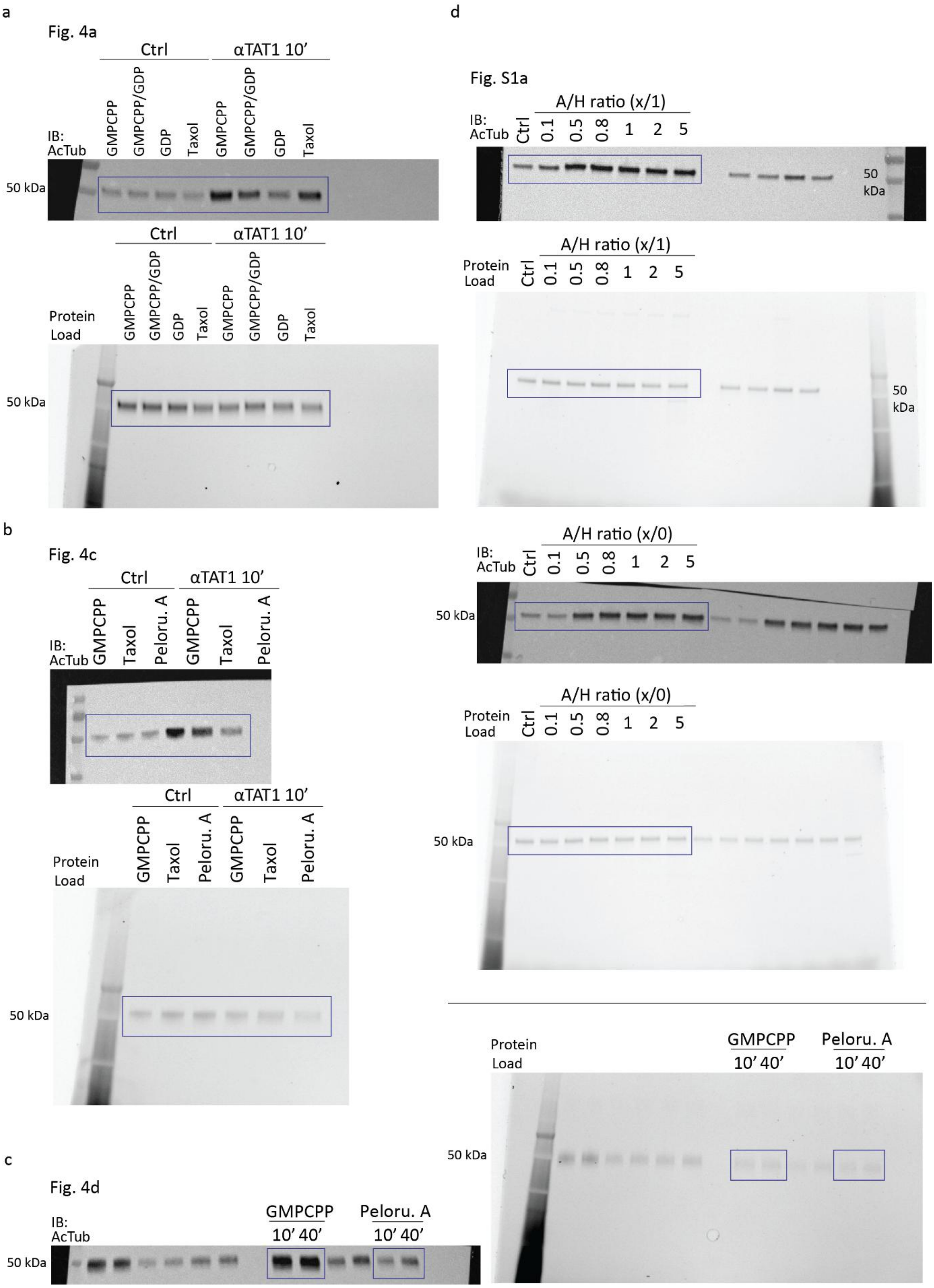

Supplementary Fig. 5. Raw data related to Fig. 4 and Supplementary Figure . a-d Uncropped Western Blot images and UV-exposed gels for protein load for (**a**) Fig. 3a, (**b**) Fig. 3b, (**c**) Fig. 3c and (**d**) Fig. 3d.
