# Supplementary material for: Microtubule lattice conformation and integrity regulate α-tubulin acetylation
Source: Commun Biol. 2026 Jun 8;9:1211. doi: 10.1038/s42003-026-10157-4 (PMC13582946; doi:10.1038/s42003-026-10157-4)
Supplement: Supplementary file 3 [file 42003_2026_10157_MOESM3_ESM.pdf]

Description of Additional Supplementary File

File name: Supplementary Data
Description: All source data is provided as “Supplementary Data”.
