# Supplementary material for: Microtubule lattice conformation and integrity regulate α-tubulin acetylation
Source: Commun Biol. 2026 Jun 8;9:1211. doi: 10.1038/s42003-026-10157-4 (PMC13582946; doi:10.1038/s42003-026-10157-4)
Supplement: Supplementary file 4 [file 42003_2026_10157_MOESM5_ESM.pdf]

nature portfolio

Corresponding author(s): Charlotte Aumeier

Last updated by author(s): Apr 9, 2026

Reporting Summary

Nature Portfolio wishes to improve the reproducibility of the work that we publish. This form provides structure for consistency and transparency in reporting. For further information on Nature Portfolio policies, see our Editorial Policies and the Editorial Policy Checklist.

Statistics

For all statistical analyses, confirm that the following items are present in the figure legend, table legend, main text, or Methods section.

n/a	Confirmed	
☐	☒	The exact sample size (*n*) for each experimental group/condition, given as a discrete number and unit of measurement
☐	☒	A statement on whether measurements were taken from distinct samples or whether the same sample was measured repeatedly
☐	☒	The statistical test(s) used AND whether they are one- or two-sided<br>*Only common tests should be described solely by name; describe more complex techniques in the Methods section.*
☒	☐	A description of all covariates tested
☒	☐	A description of any assumptions or corrections, such as tests of normality and adjustment for multiple comparisons
☐	☒	A full description of the statistical parameters including central tendency (e.g. means) or other basic estimates (e.g. regression coefficient) AND variation (e.g. standard deviation) or associated estimates of uncertainty (e.g. confidence intervals)
☐	☒	For null hypothesis testing, the test statistic (e.g. *F*, *t*, *r*) with confidence intervals, effect sizes, degrees of freedom and *P* value noted<br>*Give P values as exact values whenever suitable.*
☒	☐	For Bayesian analysis, information on the choice of priors and Markov chain Monte Carlo settings
☒	☐	For hierarchical and complex designs, identification of the appropriate level for tests and full reporting of outcomes
☒	☐	Estimates of effect sizes (e.g. Cohen's *d*, Pearson's *r*), indicating how they were calculated

Our web collection on statistics for biologists contains articles on many of the points above.

Software and code

Policy information about availability of computer code

Data collection	SlideBook 6X64 software (Version 6.0.4) was used for image acquisition in TIRF. Thermo Fisher EPU (Version 2.14) was used for transmission electron microscope image acquisition. EvolutionCapt Advance (Version 17.04) was used for chemiluminescence and EvolutionCapt FX6 (Version 17.03) for Stain-Free protein load detection.
Data analysis	Microsoft®Excel® (Version 16.0.19127.20192) and GraphPad Prism (Version 10.4.1) software were used for data analysis, statistics and the generation of graphs. ImageJ (Fiji) (Version 1.8.0_322) was used for microscopy and Western blot analysis. Adobe Illustrator (Version 29.8.1) was used to assemble the figures.

For manuscripts utilizing custom algorithms or software that are central to the research but not yet described in published literature, software must be made available to editors and reviewers. We strongly encourage code deposition in a community repository (e.g. GitHub). See the Nature Portfolio guidelines for submitting code & software for further information.

April 2023

Data

Policy information about availability of data

All manuscripts must include a data availability statement. This statement should provide the following information, where applicable:

- Accession codes, unique identifiers, or web links for publicly available datasets
- A description of any restrictions on data availability
- For clinical datasets or third party data, please ensure that the statement adheres to our policy

All data associated with this study are presented in the manuscript in main figures and the supplementary information. Source data are provided with the paper.

Research involving human participants, their data, or biological material

Policy information about studies with human participants or human data. See also policy information about sex, gender (identity/presentation), and sexual orientation and race, ethnicity and racism.

Reporting on sex and gender	n/a
Reporting on race, ethnicity, or other socially relevant groupings	n/a
Population characteristics	n/a
Recruitment	n/a
Ethics oversight	n/a

Note that full information on the approval of the study protocol must also be provided in the manuscript.

Field-specific reporting

Please select the one below that is the best fit for your research. If you are not sure, read the appropriate sections before making your selection.

☒ Life sciences ☐ Behavioural & social sciences ☐ Ecological, evolutionary & environmental sciences

For a reference copy of the document with all sections, see nature.com/documents/nr-reporting-summary-flat.pdf

Life sciences study design

All studies must disclose on these points even when the disclosure is negative.

Sample size	No statistical methods were used to predetermine sample size. Sample sizes are indicated in the figure legends.
Data exclusions	No data were excluded from the analysis.
Replication	Data were performed minimum in triplicate with similar and consistent results.
Randomization	For all experiments, control and experimental condition (aTAT1 and/or HDAC6) were treated in parallel. During imaging, microtubules of interest were randomly acquired.
Blinding	There was no blinding during data acquisition.

Reporting for specific materials, systems and methods

We require information from authors about some types of materials, experimental systems and methods used in many studies. Here, indicate whether each material, system or method listed is relevant to your study. If you are not sure if a list item applies to your research, read the appropriate section before selecting a response.

Materials & experimental systems

n/a	Involved in the study
☐	☒ Antibodies
☒	☐ Eukaryotic cell lines
☒	☐ Palaeontology and archaeology
☒	☐ Animals and other organisms
☒	☐ Clinical data
☒	☐ Dual use research of concern
☒	☐ Plants

Methods

n/a	Involved in the study
☒	☐ ChIP-seq
☒	☐ Flow cytometry
☒	☐ MRI-based neuroimaging

Antibodies

Antibodies used	Primary antibodies:<br>anti-acetylated tubulin (Sigma-Aldrich, T74451, mouse, 1:1000 dilution), anti-α-tubulin (Geneva Antibody Facility, ABCD_AA345, rabbit, 1:1000 dilution).<br><br>Secondary antibodies:<br>anti-mouse-horseradish peroxidase (HRP) (GE Healthcare, NA9310, 1:5000 dilution), species-specific IgG conjugated to Alexa-647, or 488 fluorophores (Invitrogen, 1:1000 dilution in blocking solution).
Validation	The anti-acetylated tubulin (Sigma-Aldrich, T7451) was validated for WB and ICC/IF by the manufacturer. The anti-α-tubulin (Geneva Antibody Facility, ABCD_AA345) was validated for WB and IF by the antibody facility.

Plants

Seed stocks	n/a
Novel plant genotypes	n/a
Authentication	n/a
